# Pharmacokinetics and homeostatic impact of golden bile powder: evidence from surrogate analyte-based UPLC-MS/MS in rats

**DOI:** 10.1186/s13020-026-01371-7

**Published:** 2026-04-03

**Authors:** Xin Yu, Siyang Wu, Huinan Wang, Zheng Yuan, Lifeng Zhao, Xuehao Cheng, Zhishan Huang, Qiutao Wang, Qing Zhu, Luyang Liu, Chen Kang, Baolin Bian, Yingfei Li

**Affiliations:** 1 State Key Laboratory for Quality Ensurance and Sustainable Use of Dao-di Herbs, Institute of Chinese Materia Medica, China Academy of Chinese Medical Sciences, Beijing, 100700 People’s Republic of China; 2 School of Chinese Materia Medica, Beijing University of Chinese Medicine, Beijing, 100029 People’s Republic of China; 3 Guang’an Men Hospital, China Academy of Chinese Medical Sciences, Beijing, 100053 People’s Republic of China; 4 Chongqing Jize Biotechnology Co., Ltd., Chongqing, 400700 People’s Republic of China; 5 National Resource Center for Chinese Materia Medica, China Academy of Chinese Medical Sciences, Beijing, 100700 People’s Republic of China

**Keywords:** Bile acids, Golden bile powder, Pharmacokinetics, Surrogate analyte, Endogenous bile acids, Exogenous bile acids

## Abstract

**Background:**

Golden bile powder (GBP), an artificial bear bile powder, is used for liver protection and contains taurine-conjugated bile acids (BAs) with broad pharmacological activities. However, its pharmacokinetics and effects on endogenous BA homeostasis are unclear.

**Methods:**

Sprague–Dawley rats received oral administration of GBP (54 mg/kg/day) combined with three deuterated taurine-conjugated BAs at fixed ratios for 7 days to facilitate separate analysis of changes in exogenous and endogenous BAs following administration. Single deuterated taurine-conjugated BA groups were also included. Blood samples were collected over a 24 h time window on days 1 and 7. We developed and validated a surrogate analyte-based UPLC-MS/MS method to quantify six BAs in rat plasma, eliminating endogenous interference. Pharmacokinetic parameters were obtained by non-compartmental analysis, and gender differences and accumulation effects were assessed using appropriate statistical comparisons.

**Results:**

The UPLC-MS/MS method demonstrated good linearity (r > 0.999), accuracy (89.1%–115%), precision (RSD < 15%), and stability (within ± 15%). Pharmacokinetic studies showed taurine-conjugated BAs in GBP had long half-lives (8.43–12.7 h) and gender-specific accumulation, with females displaying higher systemic exposure. For example, the AUC_0-24 h_ of TCA was approximately 16-fold higher in females than in males. Repeated GBP administration (54 mg/kg/day for 7 days) reshaped systemic BA composition, increasing both exogenous and endogenous BA concentrations, with the latter displaying more pronounced elevations—approximately fivefold higher than the exogenous BA AUC increase. Sex differences were evident, as female rats exhibited enhanced conversion of conjugated BAs to free forms. The BA shifts observed may be related to known enterohepatic circulation and gut microbiota-mediated BA transformation pathways.

**Conclusions:**

This work establishes an analytical framework for evaluating endogenous and exogenous BA dynamics, contributing to future mechanistic and translational studies involving GBP and its potential applications.

**Graphical Abstract:**

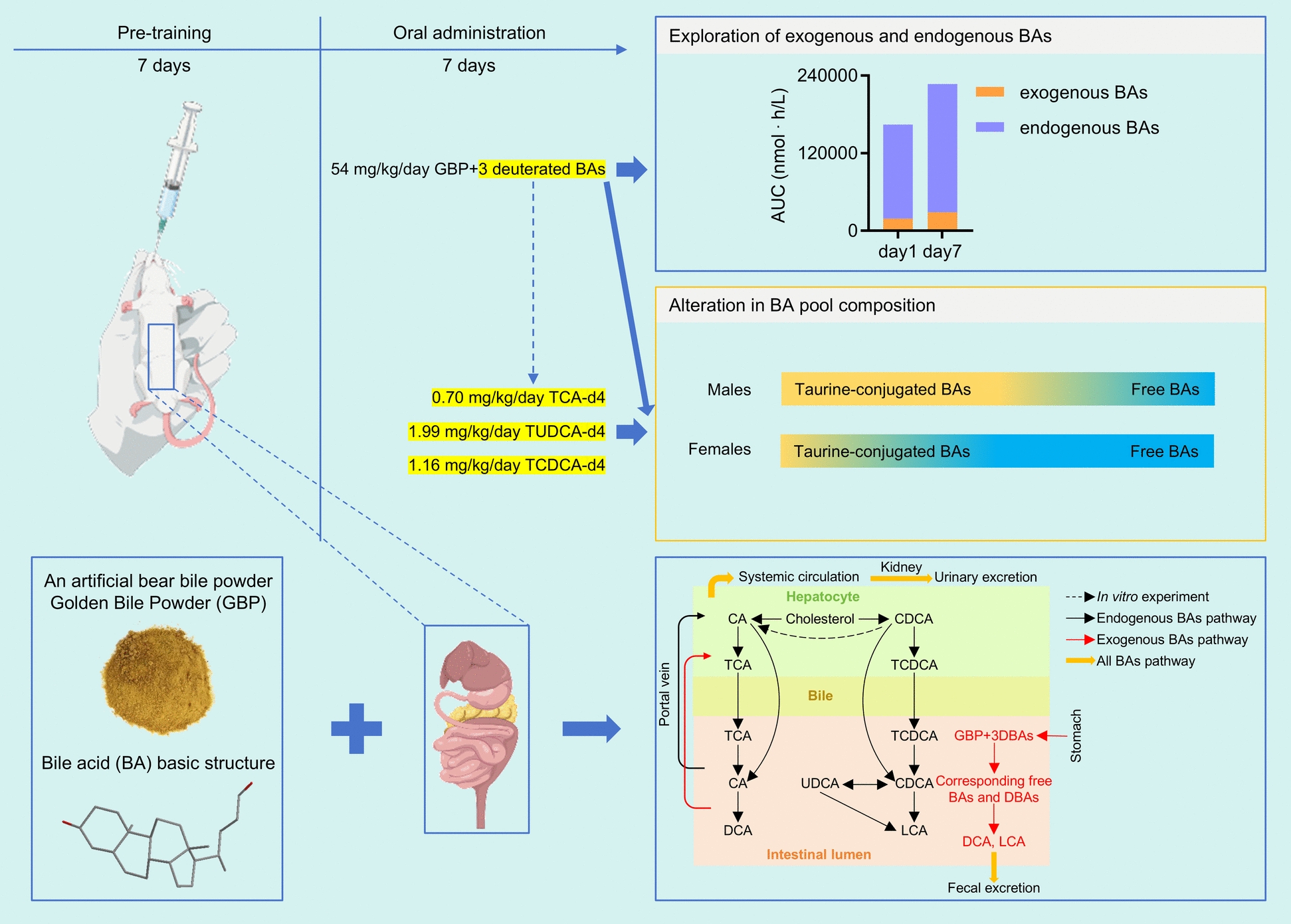

**Supplementary Information:**

The online version contains supplementary material available at 10.1186/s13020-026-01371-7.

## Introduction

Bile acids (BAs) are amphipathic molecules synthesized in hepatocytes and known as natural detergents [1, 2]. Primary BAs (cholic acid, CA; chenodeoxycholic acid, CDCA) derive from hepatic cholesterol via CYP7A1, CYP8B1, et al. mediated pathways, while secondary BAs (deoxycholic acid, DCA; lithocholic acid, LCA) reflect gut microbial transformations from primary BAs [1, 3]. Ursodeoxycholic acid (UDCA), arose from CDCA by hydroxysteroid dehydrogenase (HSDH), is a well-known BA used clinically for hepatoprotection [4]. Conjugation with taurine or glycine enhances solubility. Crucially, through receptors including FXR, TGR5, PXR and VDR, BAs regulate lipid and glucose homeostasis, modulate inflammation, and mediate liver–gut–microbiota–immune crosstalk [2, 5]. Dysregulation of BA homeostasis contributes to diverse pathologies, including cholestasis, liver fibrosis, metabolic syndrome, and inflammatory disorders [6], making BA signaling an attractive therapeutic target.

In traditional Chinese medicine, animal bile has long been used to treat a variety of ailments, with bear bile being the most renowned. Importantly, bear bile contains a relatively high content of TUDCA, while that in other animals’ bile is very low. Research indicates that TUDCA is not only safer but also more effective in various pharmaceutical applications, particularly in treating hepatobiliary diseases and improving outcomes in central nervous system disorders [7]. However, the use of natural bear bile is restricted due to ethical and conservation concerns [8]. As a response to this, artificial bear bile powder (ABBP) has been developed as a synthetic substitute that mimics the chemical composition of natural bear bile powder. Extensive studies have demonstrated that ABBP exhibits broad bioactivities, including hepatoprotective [9], anticancer [10], anti-inflammatory [11], anticonvulsant [12], hypoglycemic [13], antioxidant [14], and ophthalmic effects [15], impacting cardiovascular and central nervous systems [16].

Golden bile powder (GBP), an ABBP, is produced from chicken bile by enzymatic biotransformation that converts taurine chenodeoxycholic acid (TCDCA) to taurine ursodeoxycholic acid (TUDCA), yielding in a product chemically comparable to natural bear bile powder, with taurine-conjugated bile acids (BAs)—particularly TUDCA—as its principal active components [7]. In murine models, GBP reduces blood alcohol levels, attenuates liver steatosis, and rebalances gut microbiota. GBP promoted for mitigating alcohol induced liver injury and promoting hepatoprotection [17].

Despite these pharmacological insights, the pharmacokinetic (PK) interplay between exogenous BAs from bile powder like GBP and endogenous BA pools remains unclear. A major obstacle is to exclude dynamic endogenous BA interference for accurate quantification in biological matrices. To address this challenge, surrogate analyte strategies using stable isotope-labeled compounds, as recommended by the ICH M10 guideline, enables accurate quantification through calibration curves with deuterated analogs [18–21]. Although applied to compare pharmacokinetics of different bear bile powders, prior studies did not explicitly distinguish exogenous BA pharmacokinetics from endogenous pool [22]. This separation is essential to evaluate absorption, distribution, metabolism, excretion of exogenous BAs and their impact on endogenous BA homeostasis, and it is equally important for evaluating the therapeutic potential of ABBP, given that BA signaling is tightly regulated through enterohepatic circulation and gut microbiota–mediated transformations.

To fill this critical gap, we developed a surrogate analyte-based UPLC-MS/MS method incorporating three deuterated taurine-conjugated bile acids (TCA-d4, TUDCA-d4, TCDCA-d4) into GBP formulations, permitting precise delineation of exogenous versus endogenous BA pharmacokinetics in rats. By administering deuterated bile acids (DBAs) in combination and individually, we capture BA specific metabolic trajectories, reveal sex dependent accumulation and metabolism patterns, and quantify how GBP intake reshapes the endogenous BA milieu. This dual fraction analytical framework not only advances mechanistic understanding of BA-derived therapeutics but also establishes a versatile platform for PK studies of endogenous bioactive compounds in drug development. Our findings provide critical insights into GBP’s systemic actions, supporting its development as a safe and sustainable alternative to natural bear bile for managing BA-related disorders. The aim of this study was to validate a surrogate-analyte UPLC-MS/MS method and apply it to distinguish exogenous and endogenous BA pharmacokinetics after GBP administration in rats using noncompartmental analysis.

## Materials and methods

### Chemicals and reagents

Taurocholic acid sodium salt (TCA sodium salt, C_26_H_44_NO_7_SNa, molecular weight (MW): 537.68, lot: 110815-201911, purity: 87.3%), TUDCA (C_26_H_45_NO_6_S, MW: 499.7, lot: 110816-202110, purity: 92.8%), UDCA (C_24_H_40_O_4_, MW: 392.57, lot: 110755-202005, purity: 99%), CDCA (C_24_H_40_O_4_, MW: 392.57, lot: 110806-202009, purity: 96.2%) were all purchased from National Institutes for Food and Drug Control. CA (C_24_H_40_O_5_, MW: 408.57, lot: E-816200-NU1, purity: 99%) was provided by CFW Laboratories, Inc. TCDCA sodium salt (C_26_H_44_NO_6_SNa, MW: 521.68, lot: 21J207-E5, purity: 99.66%), taurocholic acid-d4 sodium salt (TCA-d4 sodium salt, C_26_D_4_H_40_NO_7_SNa, MW: 541.72, lot: 21J177-A3, purity: 99.80%), tauroursodeoxycholic acid-d4 sodium salt (TUDCA-d4 sodium salt, C_26_D_4_H_40_NO_6_SNa, MW: 525.71, lot: 21J158-E2, purity: 100%), taurochenodeoxycholic acid-d4 sodium salt (TCDCA-d4 sodium salt, C_26_D_4_H_40_NO_6_SNa, MW: 525.71, lot: ZZS-20-057-A7, purity: 99.27%), cholic acid-d4 (CA-d4, C_24_D_4_H_36_O_5_, MW: 412.60, lot: ZZS-20-013-B9, purity: 99.32%) were all purchased from Shanghai zzbio Co., Ltd. Ursodeoxycholic acid-d4 (UDCA-d4, C_24_H_36_D_4_O_4_, MW: 396.6, lot: 1032-048A1, purity: 99.9%) was purchased from TLC Pharmaceutical Standard. Chenodeoxycholic acid-d4 (CDCA-d4, C_24_H_36_D_4_O_4_, MW: 396.6, lot: E0026997, purity: 98.7%) was purchased from Beijing Manhage Biotechnology Company. Diclofenac (Internal standard 1, IS1, C_14_H_9_Cl_2_NO, MW: 296.15, lot: PS0395, purity ≥ 99%) was purchased from Shanghai PureOne BioTech Co., Ltd. Tolbutamide (Internal standard 2, IS2, C_12_H_18_N_2_O_3_S, MW: 270.35, lot: M26M7L15275, purity ≥ 99%) was provided by Shanghai Yuanye Biotechnology Co., Ltd. GBP (lot: 20220501, containing 11.6% TCA, 33.2% TUDCA, 19.3% TCDCA) was produced by Jinxiong Pharmaceutical (Zhuhai Hengqin) Co., Ltd. The structural formula of each compound is displayed in Fig. S1.

Acetonitrile (ACN, LC–MS grade) was purchased from Honeywell (Morristown, USA). Formic acid (FA, HPLC grade) was produced by ROE company (USA). Ammonium formate (AF, LC–MS grade) was produced by Sigma-Aldrich (USA). Ultrapure water was pre-prepared through a Milli-Q ultra-pure system (Millipore, Bedford, MA, USA).

### Animals and PK study

Twenty-four Sprague–Dawley rats (200 ± 20 g) were obtained from Beijing Vital River Laboratory Animal Technology (Beijing, China; SCXK 2016 (jing)-0006). The rats were housed in a controlled environment with a temperature of 22 ± 2 ℃ and humidity of 60 ± 5%, and were provided with free access to standard laboratory diet and water. The experiment protocol was approved by the Ethics Committee of the Institute of Chinese Materia Medica, China Academy of Chinese Medical Sciences, and conducted in accordance with relevant ethical guidelines and regulations (ethical number: 2021B169).

A total of 24 rats, consisting of 12 males and 12 females, were randomly assigned to four groups, each comprising six rats (3 males and 3 females). The groups were as follows: GBP + 3DBAs (54 mg/kg/day GBP, 0.70 mg/kg/day TCA-d4, 1.99 mg/kg/day TUDCA-d4, 1.16 mg/kg/day TCDCA-d4), TCA-d4 (0.70 mg/kg/day), TUDCA-d4 (1.99 mg/kg/day), and TCDCA-d4 (1.16 mg/kg/day). The solution is prepared with water, and the dosage volume is 10 mL/kg. Prior to drug administration, the rats were fasted for 18 h while being allowed ad libitum access to water. GBP and DBAs was dissolved in water and administered orally via gavage. All animals in each group received continuous administration for 7 days. After rats were anesthetized by inhaling isoflurane, blood samples (200 μL) were collected at predetermined time points: 0, 0.17, 0.5, 1, 2, 4, 6, 8, 12 and 24 h post-administration on day 1 and 7. The total daily blood collection shall not exceed 2 mL. These samples were drawn into heparin-coated tubes and immediately centrifuged at 12000 rpm for 2 min to separate plasma. The resulting plasma samples were then stored at − 70 °C until analysis. Samples showing visible red discoloration were considered hemolyzed and were not included in further analysis. And no sample met the exclusion criteria for hemolysis in this experiment. All rats were euthanized with CO_2_ after use. The plasma concentration–time data were subjected to noncompartmental analysis using Phoenix WinNonlin 8.2 software (Pharsight, Mountain View, CA, USA) to calculate PK parameters.

The statistical analysis and graphical representation of findings was to be conducted using GraphPad Prism 9.5 software. All parameters were assumed to describe a normal standard distribution and are expressed as mean ± standard error of the mean (SEM). Comparisons between day 1 and day 7 within the same animals were performed using paired two-tailed Student’s t-tests, whereas comparisons between male and female rats were conducted using two-tailed unpaired t-tests with unequal variance (Welch’s t-test). Statistical analyses were performed on summary PK parameters rather than at individual time points. Given the small sample size, statistical results were interpreted cautiously with emphasis on effect size and directionality. *P* < 0.05 was considered indicative of a potential difference.

### UPLC-MS/MS for PK study

An AB Sciex API 5500 Q-Trap mass spectrometer (Toronto, Canada), equipped with an electrospray ionization (ESI) source and interfaced with a Waters Acquity UPLC separation module, was used for detection and quantification of the six BAs and their corresponding six DBAs. Empower 3.0 and Analyst 1.6.2 software were used to control the UPLC and mass spectrometer, respectively. Chromatographic separation was achieved on a Waters CORTECS UPLC HSS T3 (2.1 mm × 100 mm, 1.6 μm, kept at 45 °C) using mobile phase A (water–2.5 mM FA–0.1 mM AF) and mobile phase B (ACN–2.5 mM FA–0.1 mM AF). The mobile phase was delivered at 0.3 mL/min and a gradient program was used as follows: 0–1.0 min, 30% B; 1.0–3.5 min, from 35 to 38.5% B; 3.5–7.0 min, 38.5% B; 7.0–9.0 min, from 48 to 57% B; 9.0–10.0 min, 100% B; and 10.0–11.0 min, 30% B. The weak and strong washing solution is water and ACN, respectively. The autosampler temperature was set at 6 °C. The ESI source was operated in negative ion mode with optimized parameters: ion spray voltage at –4500 V, turbo spray temperature at 500 °C, nebulizer gas at 50 psi, heater gas at 50 psi and curtain gas at 35 psi. A dwell time of 50 ms was set for all analytes. The entrance potential and collision cell exit potential were set at –10 and –16 V, respectively. The precursor-to-product ion pairs used for multiple reaction monitoring of the compounds are listed in Table S1.

### Sample preparation

#### Analysis of BAs

An accurate 150 μL volume of ACN (40 ng/mL IS1) was added to 50 μL of rat plasma samples. The mixture was then vortexed for 2 min to precipitate protein, followed by sonication in an ice-water bath for 10 min. After centrifugation at 12,000 rpm for 2 min, 80 μL of the supernatant was transferred to a tube containing 120 μL of water and vortex-mixed for an additional 2 min. A 1 μL aliquot (partial loop) of this supernatant was injected into the UPLC-MS/MS system for the analysis of six BAs.

#### Analysis of DBAs

An accurate 200 μL volume of ACN (1 ng/mL IS2) was added to 50 μL of rat plasma samples. The samples were vortexed for 2 min to precipitate protein and then subjected to sonication in an ice-water bath for 10 min. Following centrifugation at 12,000 rpm for 2 min, 200 μL of the supernatant was transferred to a new centrifuge tube and evaporated to dryness using a centrifugal vacuum concentrator. The dried residue was reconstituted with 50 μL of 30% ACN and vortex-mixed for 2 min, then subjected to further sonication n an ice-water bath for 5 min. After a final centrifugation at 12,000 rpm for 2 min, 5 μL (full loop) of the supernatant was injected into the UPLC-MS/MS system for the analysis of six DBAs.

### Surrogate analyte strategy and response factor-based quantification

Because BAs are endogenous compounds that exhibit pronounced temporal fluctuations and substantial inter-individual variability in plasma, direct quantification of exogenous BAs by blank subtraction is not appropriate. Therefore, a surrogate analyte strategy was employed, in which stable isotope-labeled analytes that are regarded as surrogate standards to construct the calibration curves for quantifying the corresponding authentic analytes. These surrogate analytes share identical chemical structures with their unlabeled analytes except for isotopic substitution. However, isotope standards may exhibit minor differences in retention time and mass spectrometric response. Accordingly, a response factor (RF) is applied for correction, defined as the ratio of the peak area response of the surrogate analyte to that of its corresponding authentic analyte at the same concentrations under identical analytical conditions. Ideally, each RF should be close to unity and remain constant across the entire calibration range. When deviations from unity are observed, the RF is incorporated into the regression equation of the calibration curve. The authentic analyte concentration (*c*_BAs_) is then calculated as follows: *c*_BAs_ = (Area Ratio_BAs_ × RF–b)/a, where a and b are the intercept and slope of the calibration curve, respectively.

### Calibration standards and quality control samples

Stock solutions for six BAs, six DBAs and two ISs were prepared at a concentration of 1 mg/mL in methanol. Subsequent working solutions were generated by serial dilution with 50% ACN and stored at –70 °C. Calibration standards and quality control (QC) samples were prepared by adding 5 μL of the working solution to 45 μL of blank rat plasma, followed by thorough mixing at 1,500 rpm for 2 min. For the analysis of six BAs, the calibration standard samples of TCA-d4 and CA-d4 were prepared across a concentration range of 20–14580 ng/mL. The QC samples were prepared at concentrations of 60, 540, and 11664 ng/mL. The calibration standard samples for the remaining DBAs were prepared within a concentration range of 10–7290 ng/mL, with QC samples prepared at 30, 270 and 5832 ng/mL. In the analysis of six DBAs, the calibration standard samples were made within a concentration range of 0.3–243 ng/mL and the QC samples were prepared at 1, 9 and 194.4 ng/mL. Two ISs (IS1 and IS2) were applied to different analyte classes and concentration ranges to ensure appropriate signal normalization across BA and DBA analyses.

### Calculation of RF for BAs and DBAs in rat plasma

A mixed working solution containing six BAs and their corresponding DBAs was prepared at two concentration levels: low and high. 5 μL of the mixed working solution was added to 45 μL of blank rat plasma and processed following the method outlined in "*Analysis of BAs*" prior to analysis. This process allowed for the acquisition of both the DBAs and BAs area ratios. Additionally, 50 μL of the same blank rat plasma was treated in the same manner and analyzed as a blank sample. The RF is calculated using the following formula: RF = Area Ratio_DBAs_/(Area Ratio_BAs_–Area Ratio_BAs in blank rat plasma_).

### Method validation

Our analytical method underwent validation in accordance with the ICH guidelines for bioanalytical method validation. The validation process encompassed assessment of the lower limit of quantification (LLOQ) and carryover, selectivity, linearity, accuracy and precision (for both surrogate and authentic analytes), matrix effects and sample stability (including both surrogate and authentic analytes). The specific validation data are provided in the Additional file 1.

## Results and discussion

### Response factor and method validation

Given that BAs are endogenous to rats, preparation of a true blank matrix is difficult. To overcome this, DBAs were applied as surrogate standards for calibration. The RF was established for each analyte, ensuring accurate quantification of TCA, TUDCA, TCDCA, CA, UDCA, and CDCA (Table S2). The RSD of RF was less than 9.79%, but the values were not close to unity. Therefore, RF should be added to the standard curve formula for calculation to accurately measure the true analyte. The stable RFs confirmed the feasibility of using DBAs for precise quantification, providing the basis for method validation.

The developed UPLC–MS/MS method was then validated to ensure selectivity, sensitivity, accuracy, precision, and stability for BA and DBA analysis in rat plasma. The method demonstrated excellent selectivity, with no significant endogenous interference or carryover (Fig. S2). Calibration curves exhibited strong linearity (r = 0.9975 ~ 0.9992) across the tested range, and sensitivity was adequate for PK evaluation (Table S3). Accuracy (89.1%–115%) and precision (RSD ≤ 11.5%) met acceptance criteria (Table S4 and S5). Matrix effects were minimal (≤ 5.98%), and analytes remained stable under different conditions, with deviations within ± 10.1% of nominal concentrations (Table S6–S8). The stock and working solutions demonstrated acceptable stability, with concentration changes remaining within ± 9.13% of initial values (Table S9 and S10). In the surrogate-analyte–based BA quantification, the true analytes, surrogate analytes, and IS1 showed consistent and mutually aligned extraction recoveries (92.0%–114%, Table S11), supporting the reliability of the substitution strategy. These results demonstrate that the method is robust and reliable, allowing us to confidently apply it to PK studies of GBP and DBAs.

### Pharmacokinetics of BAs and DBAs after combined administration

**Fig. 1:**
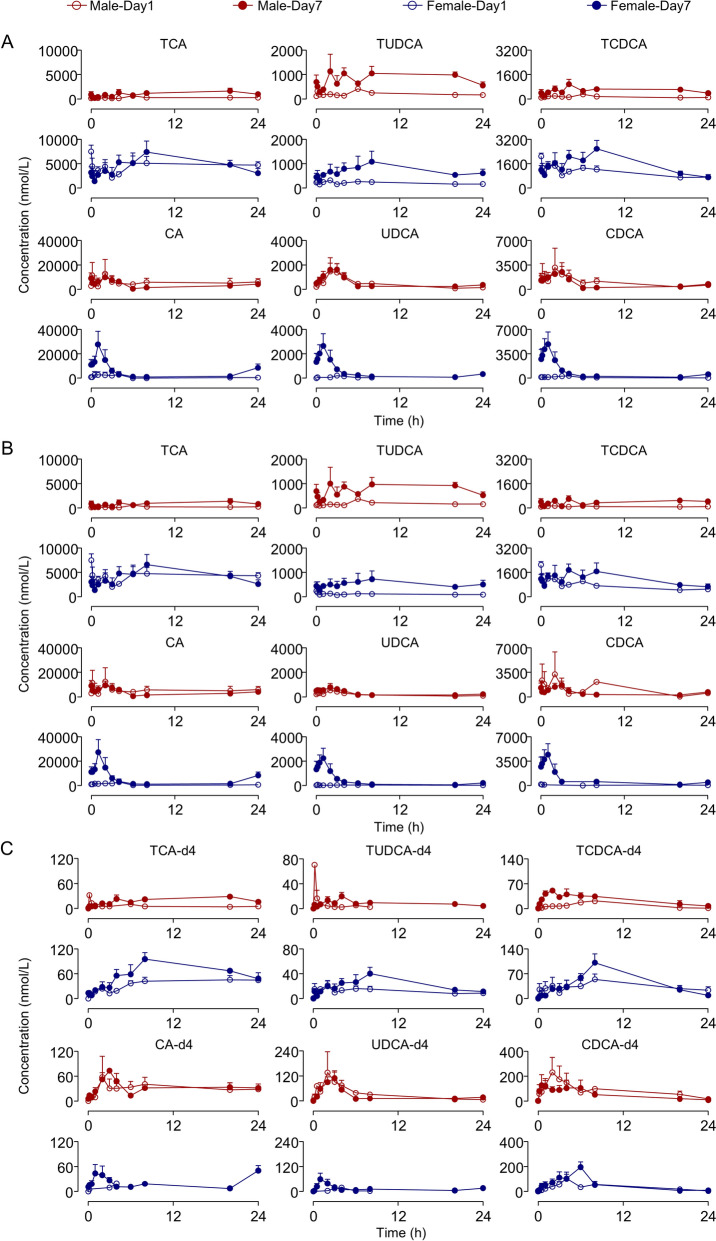
Mean plasma concentration of six BAs and six DBAs after 7-days administration of GBP + 3DBAs. Data represent mean ± SEM (*n* = 3). **A** Total BAs; **B** Endogenous BAs; **C** DBAs

Our validated analytical method effectively monitored six BAs and six DBAs in rat plasma following oral administration of GBP and DBAs. Because BAs are endogenous, PK parameters of exogenous BAs can be influenced by endogenous BAs. Consequently, we incorporated three taurine-conjugated DBAs into GBP to characterize the temporal changes of exogenous BAs in rats and to assess their impact on endogenous BAs. DBA dosage was set at one-ninth of their respective prototype in GBP, and endogenous BA levels were calculated by subtracting nine times the DBA concentration from that of the prototype. This approach assumes linearity over the relevant concentration range, comparable ADME behavior between DBAs and unlabeled BAs due to identical chemical structures except for isotopic substitution, and no meaningful interaction affecting absorption, metabolism, or enterohepatic circulation. First, given that DBAs were administered at a low dose relative to the prototypes, saturation of transporters or nonlinear disposition is low probability. In addition, the parallel temporal trends observed between DBAs and their corresponding BAs support the applicability of a proportional scaling approach for exploratory analysis. Second, stable isotope–labeled compounds are generally considered to exhibit comparable physicochemical properties and ADME behavior to their unlabeled counterparts, as isotopic substitution does not alter molecular structure or biological interactions [23]. Although potential competition at transporters, microbial conversion, or enterohepatic circulation cannot be entirely excluded, DBAs and unlabeled BAs were administered concurrently and are expected to undergo these processes synchronously within the same individual. Therefore, any such effects are unlikely to materially distort intra-animal comparisons or qualitative trends. Concentration–time profiles are depicted in Fig. 1, with *C*_max_ and AUC in Table 1, entire PK parameters in Table S12, and the accumulation indices (R_ac_) in Table 2. We calculated the AUC_0-24 h_ ratio of free to their corresponding taurine-conjugated BAs and DBAs (e.g., CA/TCA), referred to as the free/conjugated ratio (R_f/c_), and compared day 7 to day 1 values (Table 2).

**Table 1:** *C*_max_ and AUC of six BAs and six DBAs in rats after administration of GBP + 3DBAs (Mean ± SEM, *n* = 3)

PK parameters^a^	TCA		TUDCA		TCDCA		CA		UDCA		CDCA	
	♂	♀	♂	♀	♂	♀	♂	♀	♂	♀	♂	♀
Total BAs-day1												
*C*_max_ (nmol/L)	702 ± 299	7988 ± 1197^**^	554 ± 149	379 ± 66.1	339 ± 66.2	2214 ± 155^**^	18121 ± 10448	3043 ± 2626	1743 ± 1020	112 ± 57.6	3752 ± 2553	325 ± 178
AUC_0-24 h_ (nmol∙h/L)	6842 ± 1278	108991 ± 21131^*^	4958 ± 561	4967 ± 1104	3348 ± 399	23391 ± 2978^**^	131389 ± 58971	13027 ± 8677	8444 ± 2971	481 ± 133	20006 ± 9201	1794 ± 543
AUC_0-∞_ (nmol∙h/L)	9047	197698 ± 59876	7150 ± 871	7684 ± 1692	4563 ± 700	35981 ± 6201^*^	175863 ± 75198	39901	13025 ± 1888	925	30403	2789
Total BAs-day7												
*C*_max_ (nmol/L)	2106 ± 433	9442 ± 1971^*^	1628 ± 533	1642 ± 366^#^	1129 ± 264	3170 ± 430^**^	13933 ± 4082	31498 ± 7910	2024 ± 513	2739 ± 901	2882 ± 697	4967 ± 1628
AUC_0-24 h_ (nmol∙h/L)	27908 ± 8465	120280 ± 13280^**^	21311 ± 4147^**#**^	17024 ± 2562^#^	13983 ± 2285^#^	36078 ± 732^**#^	75200 ± 18232	91417 ± 15470^#^	10390 ± 2253	8557 ± 1682^#^	15614 ± 3912	15036 ± 4112
AUC_0-∞_ (nmol∙h/L)	32030 ± 10697	199439 ± 43571^*^	28681 ± 6959^#^	23218 ± 4578	19828 ± 2465^#^	49810 ± 5918^**^	67072 ± 17704	136548 ± 15089^*^	12725 ± 3384	12388 ± 1094	21063 ± 9073	19647 ± 2789^#^
Endogenous BAs-day1												
*C*_max_ (nmol/L)	616 ± 294	7972 ± 1198^**^	427 ± 140.3	243 ± 53.6	217 ± 24.3	2189 ± 165^**^	17455 ± 10182	3001 ± 2583	649 ± 358	70.1 ± 16.5	2255 ± 1566	174.5 ± 74.6
AUC_0-24 h_ (nmol∙h/L)	5469 ± 1441	100831 ± 19789^**^	4421 ± 562	2400 ± 545^*^	2553 ± 393	15687 ± 545^**^	126328 ± 57226	12808 ± 8654	3700 ± 1245	382 ± 64.8^*^	15242 ± 6784	1018 ± 563
AUC_0-∞_ (nmol∙h/L)	7406 ± 1310	151095 ± 47669^*^	6608 ± 726	4390 ± 899	3776 ± 922	21795 ± 3806^**^	171065 ± 74531	15892 ± 8345	5356 ± 2027	467 ± 56.6	21549	1068
Endogenous BAs-day7												
*C*_max_ (nmol/L)	1822 ± 403	8528 ± 1793^*^	1442 ± 509	1196 ± 277	769 ± 137^#^	2659 ± 219^**^	13443 ± 4048	30914 ± 7840	951 ± 258	2262 ± 767	2203 ± 588	4511 ± 1429
AUC_0-24 h_ (nmol∙h/L)	23380 ± 7830	106161 ± 12430^**^	19438 ± 4230^#^	12254 ± 2207^#^	8601 ± 1102^#^	26618 ± 1368^**##^	69867 ± 17251	88441 ± 15123^#^	5634 ± 1218	6515 ± 1368^#^	14112 ± 4082	14996 ± 3936^#^
AUC_0-∞_ (nmol∙h/L)	36115 ± 9809	185863 ± 28684^**^	26442 ± 6981	17854 ± 3651	9429 ± 1106	42035 ± 12440^*^	74962 ± 17255	112621 ± 20228^#^	7045 ± 2004	7241 ± 1348^#^	18577 ± 3062	19951 ± 5447

	TCA-d4		TUDCA-d4		TCDCA-d4		CA-d4		UDCA-d4		CDCA-d4	
DBAs-day1												
*C*_max_ (nmol/L)	15.7 ± 5.54	53.5 ± 8.86^*^	21.7 ± 16.3	29.7 ± 6.05	22.3 ± 4.74	58.5 ± 18.5	59.0 ± 23.9	7.50 ± 3.86	123 ± 72.9	10.8	214 ± 114	129 ± 44.2
AUC_0-24 h_ (nmol∙h/L)	114 ± 23.4	900 ± 159^*^	35.2 ± 7.99	283 ± 68.4^*^	173 ± 58.9	834 ± 286	508 ± 114	10.6 ± 10.3^*^	543 ± 195	30.2	1564 ± 478	732 ± 213
AUC_0-∞_ (nmol∙h/L)	131	1473	49.7 ± 12.0	424 ± 77.5^*^	243	1333 ± 577	655	–	803 ± 104	–	1654 ± 513	764 ± 216
DBAs-day7												
*C*_max_ (nmol/L)	31.4 ± 3.93	107 ± 16.3^*^	24.2 ± 5.76	50.5 ± 9.10	59.9 ± 9.63^#^	102 ± 23.8	60.9 ± 12.8	63.9 ± 10.7^#^	130 ± 30.7	64.3 ± 24.2	185 ± 63.2	197 ± 42.2
AUC_0-24 h_ (nmol∙h/L)	502 ± 70.4^#^	1552 ± 108^**#^	200 ± 25.7^##^	519 ± 59.1^**#^	565 ± 170	802 ± 101	641 ± 126	309 ± 21.6^##^	528 ± 125	269 ± 66.5	1253 ± 481	1126 ± 278
AUC_0-∞_ (nmol∙h/L)	1009 ± 395^#^	2597 ± 434^*^	354 ± 78.8^#^	756 ± 180	680 ± 272	940 ± 109	871 ± 396	768 ± 109	653 ± 137	344 ± 95.2	1318 ± 496	1235 ± 372

^a^*C*_max_, peak concentration; *T*_max_, time to peak concentration; AUC_0-24 h_, area under the curve from 0 to 24 h; AUC_0-∞_, area under the curve from 0 extrapolated to infinite time. ^*^P < 0.05, ^**^P < 0.01 *vs.* Male/Female; ^#^*P* < 0.05, ^##^*P* < 0.01 *vs.* Day 1/Day 7; –, data cannot be calculated, because half-lives were not estimable

**Table 2:** R_ac_ of analytes and the day 7/day 1 ratios of R_f/c_ in GBP + 3DBAs group

Total BAs			Endogenous BAs		DBAs		
Compound	♂	♀	♂	♀	Compound	♂	♀
R_ac_							
TCA	4.62	1.24	5.04	1.19	TCA-d4	5.38	1.88
TUDCA	4.27	4.29	4.33	7.34	TUDCA-d4	6.47	2.13
TCDCA	4.44	1.61	3.73	1.71	TCDCA-d4	4.06	1.43
CA	1.50	23.4	1.42	25.7	CA-d4	1.31	790^a^
UDCA	1.81	22.6	2.21	21.1	UDCA-d4	1.32	18.5
CDCA	1.49	16.2	1.39	15.9	CDCA-d4	2.00	2.59
The ratios of R_f/c_ on day 7 to day 1							
CA/TCA	0.32	23.6	0.30	27.6	CA-d4/TCA-d4	0.29	566^a^
UDCA/TUDCA	0.43	8.04	0.51	6.51	UDCA-d4/TUDCA-d4	0.25	7.45
CDCA/TCDCA	0.35	9.51	0.38	9.12	CDCA-d4/TCDCA-d4	0.38	5.28

^a^The extreme ratio arises due to a very small denominator. Following administration on day 1, the AUC of CA-d4 in female rat plasma were low

As demonstrated by the concentration–time profiles, taurine-conjugated DBAs can rapidly metabolize to free DBAs, consistent with taurine-conjugated BAs. The multi-peak phenomenon and extended half-life reflected enterohepatic circulation and the influence of circadian rhythm. The PK parameters of prototypic BAs demonstrate the transepithelial variation of BAs as a whole in rats. *T*_max_ of taurine-conjugated BAs in the GBP + 3DBAs group reached 17.0 h, indicating slow absorption. *C*_max_ and AUC revealed marked sex differences, with females showing higher exposure to taurine-conjugated BA (up to ~ 16-fold). Long *t*_1/2_ and MRT indicate slow elimination and prolonged retention; however, these parameters should be interpreted cautiously given the complex terminal profiles. After continuous gavage for 7 days, the AUC of BAs increased relative to day 1 (R_ac_ > 1.25) [24]. In male rats, the ratios of R_f/c_ (CA/TCA, UDCA/TUDCA and CDCA/TCDCA) on day 7 to day 1 were significantly < 1, suggesting a tendency for the BA composition to increase in taurine-conjugated BAs after continuous administration. Conversely, the ratios of in female rats were significantly > 1, suggesting an inclination towards an increase in free BAs.

**Fig. 2:**
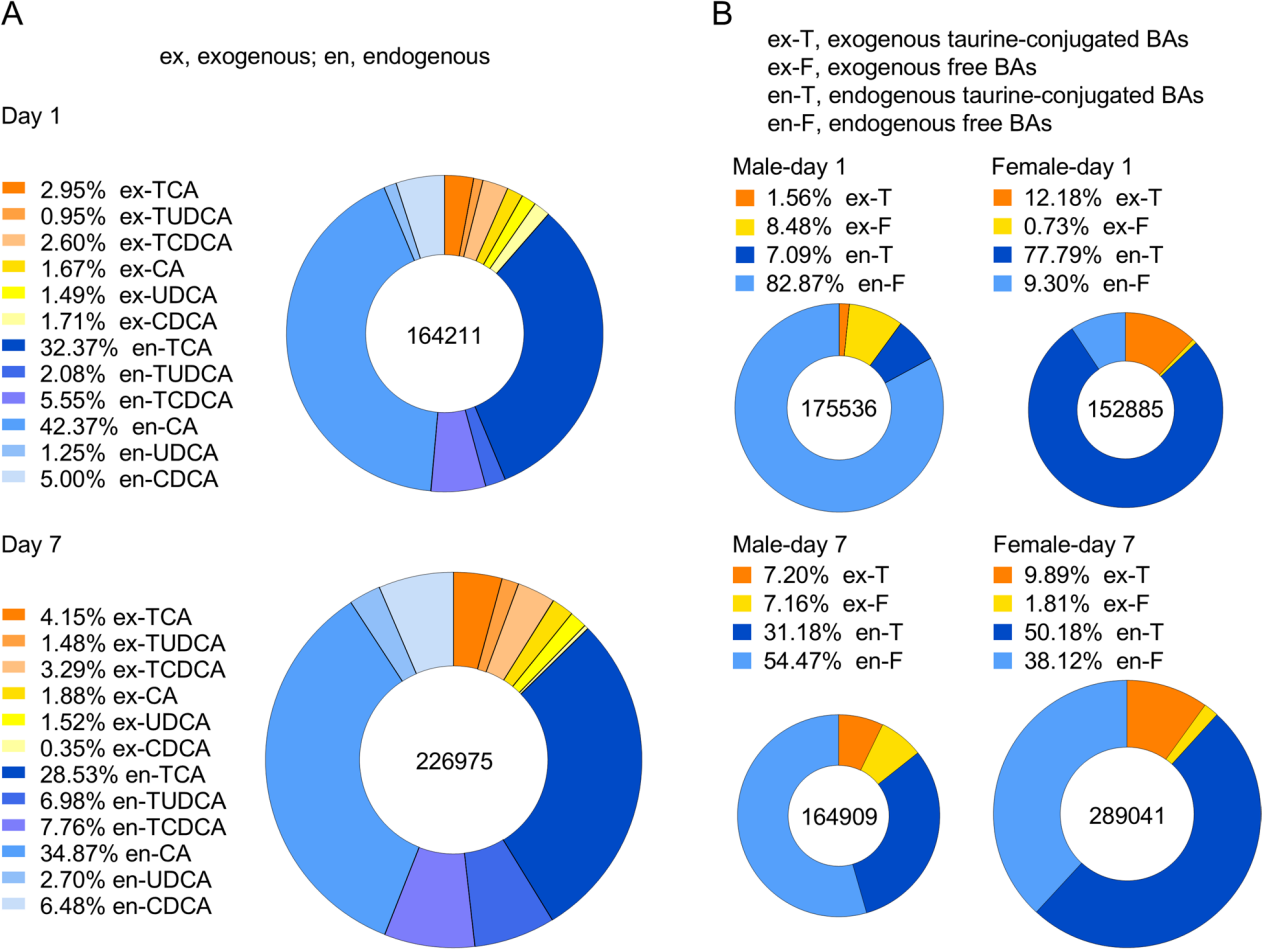
AUC (nmol∙h/L) percentage of exogenous and endogenous BAs in rat plasma after GBP administration. **A** Counting males and females together; **B** Separate counts of males and females. The values shown in the legend represent the mean percentage of each component’s AUC relative to the total mean AUC. The number inside the circle indicates the total mean AUC

DBAs are stable isotopes of BAs with similar physical and chemical properties; therefore, when rats are administrated DBAs and exogenous BAs concurrently, the PK characteristics of DBAs can be expected to reflect that of exogenous BAs. The *T*_max_ of DBAs was similarly long (up to 19.0 h), well beyond the gastric emptying time, supporting intestinal absorption [25, 26]. *C*_max_ and AUC showed clear sex differences for taurine-conjugated DBAs, with higher absorption in females. Most *t*_1/2_ and MRT were > 6 h, indicating that BAs are eliminated slowly and have a long residence time, which may be related to multiple metabolic reactions in vivo. Repeated dosing increased *C*_max_ and AUC significantly for all DBAs (except CDCA-d4 in males), indicating accumulation. The increase in DBAs suggests enhanced absorption of exogenous BAs. R_ac_ values, as well as increased *t*_1/2_ and MRT further attributed the observation of accumulation in the system. The ratios of DBAs’ R_f/c_ on day 7 to day 1 paralleled those of prototypical BAs, supporting the observation that the exogenous BA composition of male rats moved toward taurine-conjugated BAs after continuous administration, whereas females moved toward free BAs. Although some extreme ratios arise from very small denominators, these values do not affect the overall directional trends and should be interpreted with caution. We also investigated the variation in endogenous BAs. AUC of endogenous BAs also increased after 7 days, with greater rises than exogenous BAs (Fig. 2). Moreover, the ratios of endogenous BAs’ R_f/c_ on day 7 to day 1 mirrored those of total and exogenous BAs, indicating a consistent trend between exogenous and endogenous BAs. This combined administration revealed clear sex-dependent differences and accumulation of taurine-conjugated versus free BAs, prompting further investigation of individual BA behaviors.

### Pharmacokinetics after individual taurine-conjugated DBAs administration

**Fig. 3:**
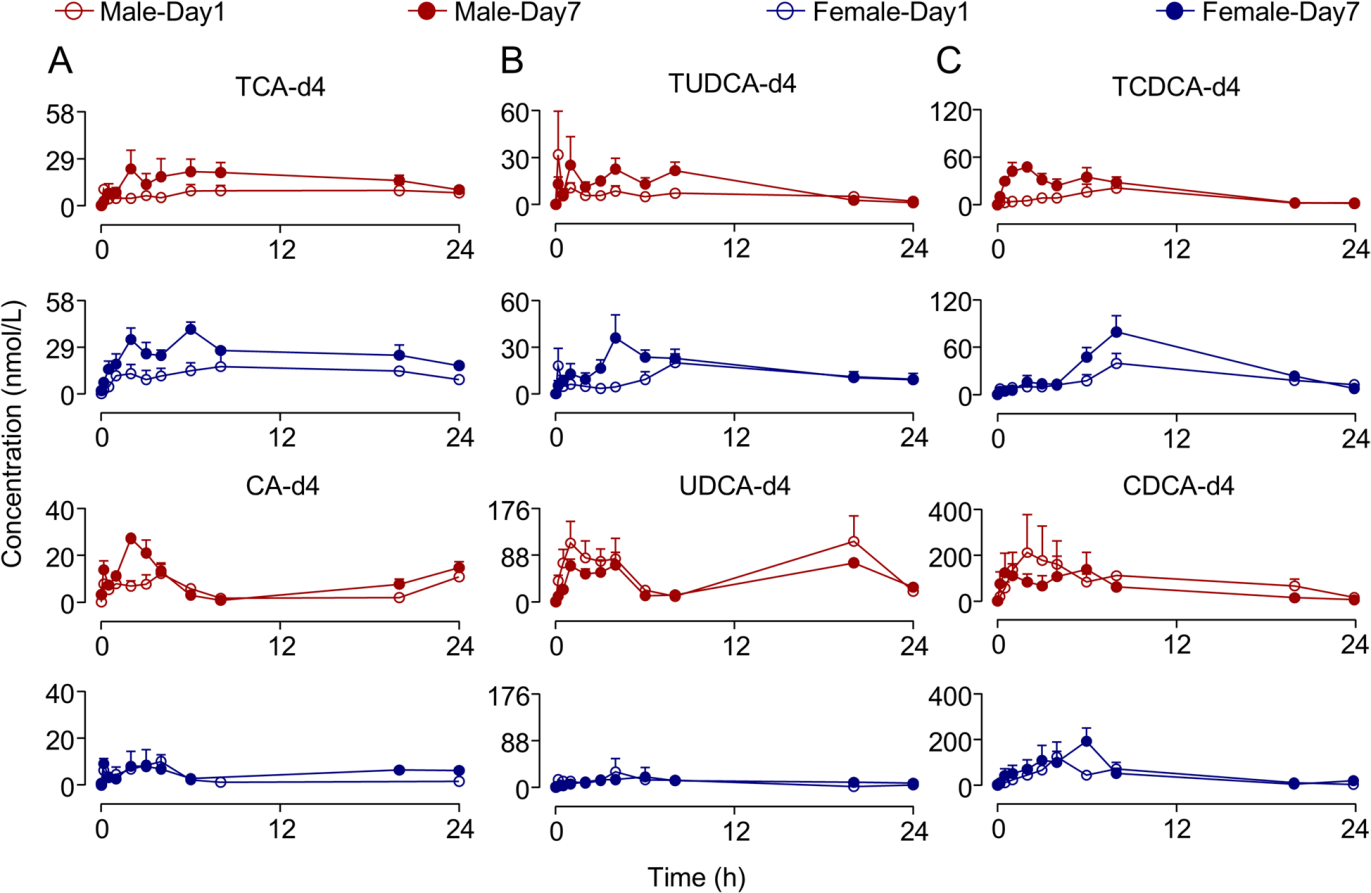
Mean plasma concentration of six DBAs in rat plasma after administration of three taurine-conjugated DBAs. Data represent mean ± SEM (*n* = 3). **A** TCA-d4 group; **B** TUDCA-d4 group; **C** TCDCA-d4 group

To comprehend temporal changes of individual taurine-conjugated BAs in vivo, we conducted three separate experiments in which rats received the same dose of TCA-d4, TUDCA-d4, or TCDCA-d4 as in the GBP + 3DBAs group. Concentration–time profiles of the six DBAs are displayed in Fig. 3, with PK parameters in Table 3, and R_ac_ values and the ratios of free/conjugated ratios on day 7 to day 1 in Table 4.

**Table 3:** PK parameters of six DBAs in rats after oral administration of three taurine-conjugated DBAs (Mean ± SEM, *n* = 3)

PK parameters^a^	TCA-d4 group				TUDCA-d4 group				TCDCA-d4 group			
	TCA-d4		CA-d4		TUDCA-d4		UDCA-d4		TCDCA-d4		CDCA-d4	
	♂	♀	♂	♀	♂	♀	♂	♀	♂	♀	♂	♀
Day 1												
*C*_max_ (nmol/L)	11.9 ± 3.39	22.5 ± 6.33	15.5 ± 2.54	7.12 ± 2.71	36.9 ± 25.0	23.4 ± 8.34	149 ± 48.1	36.5 ± 22.6	21.8 ± 6.78	39.2 ± 12.7	271 ± 139	158 ± 46.1
*T*_max_ (h)	9.06 ± 7.50	9.67 ± 5.55	9.67 ± 7.20	0.78 ± 0.61	1.72 ± 1.16	10.7 ± 7.01	13.7 ± 6.33	12.0 ± 6.11	7.33 ± 0.67	8.00 ± 0.00	5.33 ± 1.76	5.33 ± 1.33
AUC_0-24 h_ (nmol∙h/L)	198 ± 66.6	319 ± 68.5	88.1 ± 3.95	30.3 ± 12.4^*^	140 ± 12.0	281 ± 55.3	1407 ± 394	196 ± 78.4	155 ± 77.4	505 ± 129	1970 ± 210	897 ± 148^*^
AUC_0-∞_ (nmol∙h/L)	263	478	152 ± 54.1	31.8 ± 12.6	169 ± 13.4	387 ± 101	1472 ± 393	272 ± 43.3	183 ± 67.2	689 ± 215	2048 ± 236	924 ± 155^*^
*t*_1/2_ (h)	3.65	6.61	4.92 ± 3.00	0.84 ± 0.27	9.77 ± 0.99	7.91 ± 2.96	2.02 ± 0.63	15.4 ± 11.6	3.19 ± 0.19	9.28 ± 1.38^*^	2.97 ± 1.18	3.95 ± 0.20
MRT (h)	12.9 ± 0.11	12.8 ± 0.71	9.77 ± 1.28	7.39 ± 4.03	10.5 ± 0.83	12.9 ± 0.68	13.9 ± 1.54	9.66 ± 1.70	7.88 ± 1.28	11.9 ± 0.84	9.65 ± 2.21	8.08 ± 1.76
Day 7												
*C*_max_ (nmol/L)	26.3 ± 9.90	41.0 ± 5.10	27.8 ± 2.49^##^	14.3 ± 3.80^*#^	38.4 ± 12.1	36.1 ± 14.6	90.4 ± 4.10	26.4 ± 13.9^*^	50.4 ± 7.27	80.0 ± 19.6	153 ± 77.8	197 ± 59.6
*T*_max_ (h)	9.33 ± 5.46	4.67 ± 1.33	2.33 ± 0.33	1.11 ± 0.94	2.67 ± 0.88	4.67 ± 0.67	3.00 ± 1.00	10.0 ± 5.03	3.00 ± 1.53	7.33 ± 0.67	2.83 ± 1.64	5.00 ± 1.00
AUC_0-24 h_ (nmol∙h/L)	395 ± 110	602 ± 67.4	177 ± 22.1	114 ± 12.8^##^	216 ± 70.3	385 ± 120	1013 ± 150	253 ± 88.2^*^	382 ± 86.4	811 ± 97.0^*^	1235 ± 671	1126 ± 393
AUC_0-∞_ (nmol∙h/L)	501 ± 187	824 ± 29.5	196 ± 22.3	122 ± 12.7^##^	227 ± 65.6	567 ± 240	1055 ± 157	338	388 ± 87.1	867 ± 97.6^*^	1286 ± 689	1213 ± 378
*t*_1/2_ (h)	5.39 ± 4.01	10.3 ± 5.15	0.98 ± 0.16	1.00 ± 0.15	2.80 ± 0.74^#^	11.3 ± 3.08	1.09 ± 0.18	4.15	3.21 ± 0.37	5.23 ± 0.56^*#^	6.37 ± 2.12	3.52 ± 0.58
MRT (h)	12.2 ± 1.09	11.6 ± 0.69	12.2 ± 0.76	15.0 ± 2.88	6.74 ± 1.31^#^	10.4 ± 0.30	14.6 ± 0.46	13.7 ± 2.39	6.50 ± 0.48	11.6 ± 0.68^**^	7.57 ± 0.16	7.91 ± 1.31

^a^*C*_max_, peak concentration; *T*_max_, time to peak concentration; AUC_0-24 h_, area under the curve from 0 to 24 h; AUC_0-∞_, area under the curve from 0 extrapolated to infinite time; *t*_1/2_, elimination half-life; MRT, mean residence time. ^*^P < 0.05, ^**^P < 0.01 *vs.* Male/Female; ^#^*P* < 0.05, ^##^*P* < 0.01 *vs.* Day 1/Day 7

**Table 4:** R_ac_ of six DBAs and R_f/c_ in other groups

R_ac_		♂	♀	R_f/c_	♂			♀		
					Day 1	Day 7	Day 7/Day 1	Day 1	Day 7	Day 7/Day 1
TCA-d4 group	TCA-d4	2.24	2.22	CA-d4/TCA-d4	0.53	0.60	1.13	0.12	0.19	3.48
	CA-d4	2.04	3.23							
TUDCA-d4 group	TUDCA-d4	1.64	1.31	UDCA-d4/TUDCA-d4	9.80	7.66	0.69	0.84	0.80	3.11
	UDCA-d4	0.78	3.71							
TCDCA-d4 group	TCDCA-d4	3.58	1.94	CDCA-d4/TCDCA-d4	20.9	4.69	0.16	1.87	1.41	0.81
	CDCA-d4	0.72	1.24							

The profiles of DBAs in the single-compound groups resembled those in the GBP + 3DBAs group. After 7 days of dosing, AUCs of all DBAs except UDCA-d4 and CDCA-d4 increased compared to day 1 (R_ac_ > 1.25). In females, the ratios of free/conjugated ratios of CA-d4/TCA-d4 and UDCA-d4/TUDCA-d4 on day 7 to day 1 were > 1, indicating an increase in free BAs in the BA composition after continuous administration, while that of CDCA-d4/TCDCA-d4 was opposite. In males, UDCA-d4/TUDCA-d4 and CDCA-d4/TCDCA-d4 ratios were < 1, suggesting an increase in taurine-conjugated BAs, while CA-d4/TCA-d4 ratio was reversed. This inference differs slightly from those observed in the GBP + 3DBAs group. Notably, the six DBAs could interconvert in rats (Fig. S3). Thus, PK parameters in the GBP + 3DBAs group represent combined outcomes of the three taurine-conjugated BAs and DBAs. These single-compound studies indicated interconversion among DBAs and partially confirmed the sex-specific trends observed in the combined group, prompting further assessment of their influence on the endogenous BA pool.

### Impact on endogenous BAs

Finally, we examined the influence of GBP administration on endogenous BA levels to clarify how exogenous BAs impact the endogenous BA pool in rats. Plasma concentrations of endogenous BAs are affected by circadian rhythm [27], food intake [28], and other physiological processes. Our preliminary experiments observed that BA levels were more stable in the afternoon, whereas stress from handling or blood sampling caused fluctuations. Consequently, rats were pre-trained for gavage and grasp, and all administrations and blood collections were initiated at ~ 13:00.

**Fig. 4:**
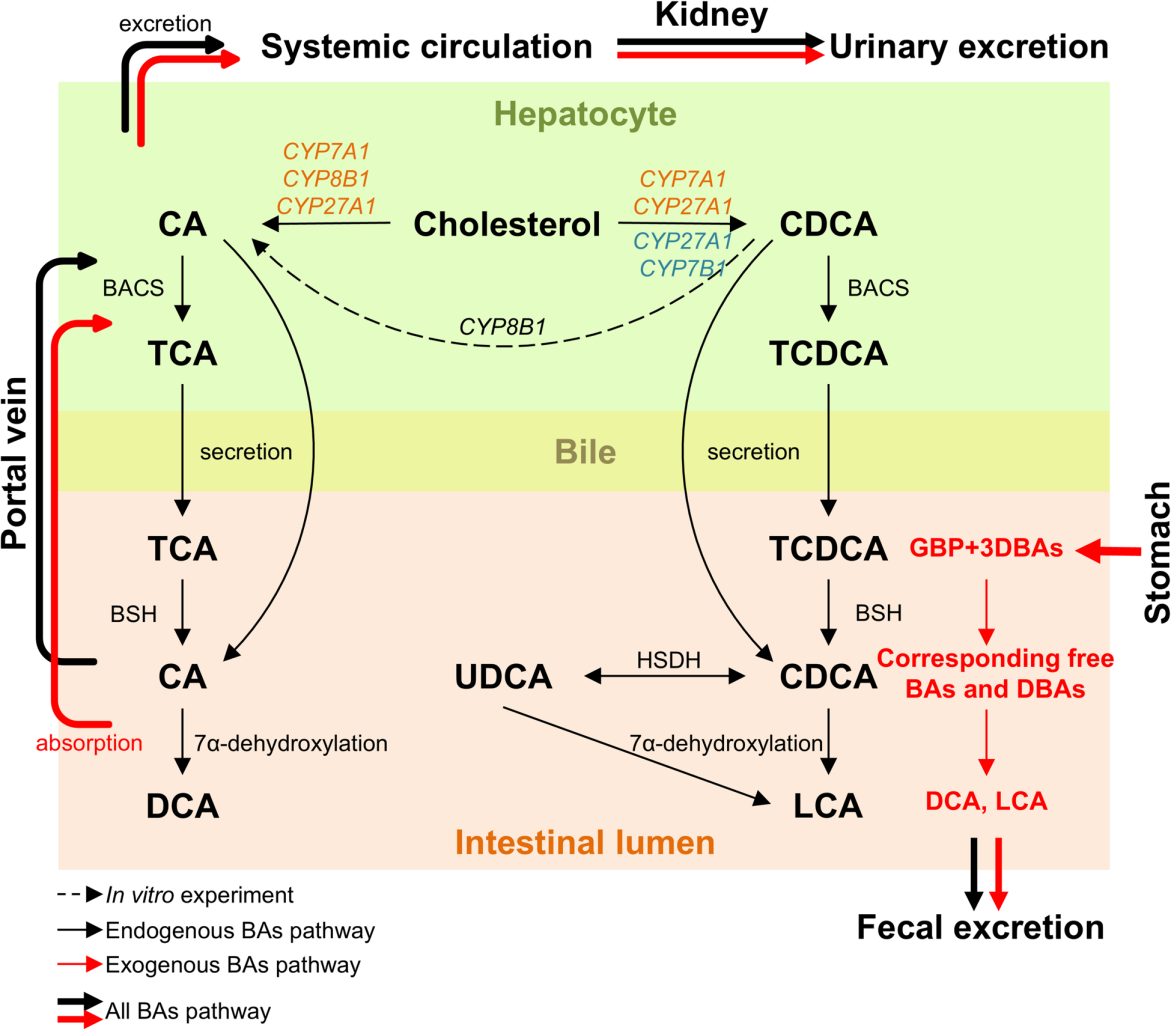
BA synthesis and metabolism pathway in hepatocytes and intestinal lumen. Endogenous BAs are synthesized in hepatocytes through the cytochrome P450 (CYP)-mediated oxidation of cholesterol, a process that occurs via the classical (orange) and alternative (blue) pathways. Within hepatocytes, the majority of BAs are conjugated to glycine or taurine (T) by the action of bile acid-CoA synthetase (BACS) and bile acid-CoA: amino acid *N*-acyltransferase before their secretion into bile via the bile salt export pump. In the intestinal lumen, glycine-conjugated and taurine-conjugated CA and CDCA are deconjugated by BSH and subsequently 7α-dehydroxylated to form secondary BAs (DCA and LCA). CDCA is further transformed into UDCA by the HSDH. Exogenous BAs are absorbed in the intestine and interact identically with endogenous BAs. At the terminal ileum, most free BAs are reabsorbed by ASBT into enterocytes and secreted into the portal circulation via the basolateral BA transporters. BAs are then taken up by NTCP and organic anion-transporting polypeptide 1 into hepatocytes. Hepatic multidrug resistance-associated protein 3 (MRP3), MRP4 and OSTα–OSTβ complex also offer alternative excretion routes for BAs into the systemic circulation. All BAs can be excreted in both urine and feces

Ingestion of exogenous BAs has been demonstrated to cause change in the BA pool, affecting both compositional ratios and overall levels. Exogenous taurine-conjugated BAs are absorbed in the intestine and deconjugated by bile salt hydrolases (BSH) from gut microbiota such as *Bacteroides*, *Clostridium*, *Lactobacillus*, *Bifidobacterium* and *Listeria*, yielding CA, UDCA and CDCA (Fig. 4). Repeated administration increased the AUC not only of exogenous but also of endogenous BAs, the latter rising more substantially. Mechanistically, exogenous BAs can modulate enterohepatic circulation by affecting BA transporters in the intestine and liver [29]. Their oral intake raises ileal luminal BA concentrations, enhancing reabsorption via the apical sodium-dependent bile acid transporter (ASBT) [30]. This transporter is primarily responsible for active BA uptake into enterocytes and plays a key role in recycling BAs back to the liver through the portal vein [31]. This process promotes recycling of both exogenous and endogenous BAs. I In addition, intestinal BA load can upregulate transporters such as organic solute transporter α/β (OSTα/OSTβ) in the basolateral membrane of enterocytes and sodium taurocholate cotransporting polypeptide (NTCP) in hepatocytes [30], further increasing recirculation efficiency (Fig. 4). Collectively, these mechanisms may contribute to expansion of the BA pool and prolong systemic retention, consistent with the observed AUC elevation of endogenous BAs after exogenous administration. Sex-specific trends may result from differences in BA-related genes expression [28] and gut microbial levels [32]. The parallel changes between exogenous and endogenous BAs indicate that GBP intake is associated with alterations in overall BA homeostasis, linking external supplementation to endogenous metabolic regulation.

This exploratory study characterized the temporal profiles of endogenous and exogenous BAs in rats following GBP administration, but several limitations should be noted. First, the distinction between endogenous and exogenous fractions was based on proportional assumptions derived from the administered dose, which may not fully reflect in vivo behavior and could introduce minor bias. The estimation of endogenous BA exposure is based on a predefined scaling factor, which may introduce uncertainty in absolute AUC values. However, sensitivity analysis using alternative scaling factors (8 ×  ~ 10 ×) showed that the overall exposure trends remained consistent (Table S13). Second, the circadian rhythm of BA secretion resulted in secondary rises at late sampling points, limiting reliable estimation of terminal-phase parameters such as half-lives and AUC_0-∞_ in some profiles. Even with an extended sampling, continued secondary rises or plateauing concentrations may occur rather than a clear terminal decline, making estimation of elimination parameters inherently challenging. Thus, the present 0–24 h sampling scheme captures exposure trends but may not fully support terminal-phase modeling for all BAs. Third, the small sample size increases the sensitivity of certain exposure ratios to near-zero denominators, potentially exaggerating apparent fold-differences; such values should therefore be interpreted cautiously. Finally, direct mechanistic assessments were beyond the scope of this study, and future work will be needed to clarify the underlying pathways.

## Conclusions

This study developed a surrogate analyte-based UPLC-MS/MS methodology that overcomes the critical challenge of accurate quantification for BAs in complex biological matrices. By strategically incorporating deuterated taurine-conjugated BAs (TCA-d4, TUDCA-d4, TCDCA-d4) into GBP, we achieved concurrent quantification and pharmacokinetic tracking of both exogenous and endogenous BA fractions in rats. Our design, including individual BA dosing and sex-based comparisons, enabled detailed PK and mechanistic insights. The validated method demonstrated high sensitivity, specificity, and reliability for BA profiling despite high endogenous background. Using this approach, we demonstrated that the exogenous taurine-conjugated BAs in GBP are slowly absorbed, accumulate with repeated dosing, leading to pronounced shifts in systemic BA profile. Strikingly, GBP intake not only raised circulating levels of exogenous BAs, but also markedly elevated endogenous BA concentrations, indicating feedback effects on enterohepatic circulation and BA synthesis. Sex-dependent differences in BA kinetics were also observed, highlighting the importance of considering sex in BA research.

Overall, this study provides important new insights into the pharmacokinetics and biological impact of GBP. It provides preliminary support for GBP as a potential alternative to natural bear bile powder and offers insights that may inform future development of therapeutics targeting BA-related disorders. Furthermore, the methodological framework developed here provides a reference for future PK studies of endogenous bioactive compounds.

## Supplementary Information

Additional file 1.

## Data Availability

The datasets used and/or analyzed during the current study are available from the corresponding author on reasonable request.

## Abbreviations

ABBP: Artificial bear bile powder
ACN: Acetonitrile
AF: Ammonium formate
ASBT: Apical sodium-dependent bile acid transporter
GBP: Golden bile powder
BA: Bile acid
BACS: Bile acid-CoA synthetase
BSH: Bile salt hydrolases
CA: Cholic acid
CDCA: Chenodeoxycholic acid
DBA: Deuterated bile acid
DCA: Deoxycholic acid
ESI: Electrospray ionization
FA: Formic acid
HSDH: Hydroxysteroid dehydrogenase
IS: Internal standard
LCA: Lithocholic acid
LLOQ: Lower limit of quantification
MRP3: Multidrug resistance-associated protein 3
NTCP: Sodium taurocholate cotransporting polypeptide
OSTα/OSTβ: Organic solute transporter α/β
PK: Pharmacokinetic
QC: Quality control
R_ac_: Accumulation indices
RF: Response factor
SEM: Standard error of the mean
TCA: Taurocholic acid
TCDCA: Taurine chenodeoxycholic acid
TUDCA: Taurine ursodeoxycholic acid
UDCA: Ursodeoxycholic acid
