# Supplementary material for: Pharmacokinetics and homeostatic impact of golden bile powder: evidence from surrogate analyte-based UPLC-MS/MS in rats
Source: Chin Med. 2026 Apr 3;21:109. doi: 10.1186/s13020-026-01371-7 (PMC13047830; doi:10.1186/s13020-026-01371-7)
Supplement: Supplementary file 1 — Additional file 1. [file 13020_2026_1371_MOESM1_ESM.docx]

1. Method validation

Our analytical method underwent rigorous validation in accordance with the ICH guidelines for bioanalytical method validation. The validation process encompassed assessment of the lower limit of quantification (LLOQ) and carryover, selectivity, linearity, accuracy and precision (for both surrogate and authentic analytes), matrix effects, sample stability (including both surrogate and authentic analytes), and dilution reliability. The specific validation tests are detailed below.

- 1. *Selectivity, and carryover*

Selectivity was ascertained by analyzing blank rat plasma sourced from ≥ 6 independent donors, which was using heparin for anticoagulation. In addition, one sample each of hemolyzed plasma and lipidemic plasma should be analyzed. Acceptance criteria stipulated no interfering peaks at the retention time of the DBAs and IS, or the peak area of the interfering peak is not greater than 20% of LLOQ and 5% of the IS. Carryover was appraised by injecting a blank sample following the upper limit of quantification (ULOQ) sample from the calibration curves; carryover was deemed acceptable if it was less than 20% of the LLOQ for surrogate analytes and 5% for IS.

- 1. *Linearity*

Duplicate calibration curves were constructed across three separate batches and subjected to analysis. The correlation coefficient (r), derived from the regression model that best fit the entire tested concentration range, was required to exceed 0.99. Additionally, the back-calculated calibration point concentrations had to exhibit precision and accuracy within ± 15% (LLOQ within ± 20%).

- 1. *Accuracy and precision*

The analytical method's performance in terms of intra- and inter-assay precision and accuracy was rigorously assessed using QC samples of DBAs at four distinct concentration levels: LLOQ, low quality control (LQC), medium quality control (MQC), and high quality control (HQC). This evaluation involved analyzing six replicates per concentration level across three consecutive batches. Precision, expressed as relative standard deviation (RSD%), should not exceed 15% (or 20% at LLOQ). Accuracy, defined as the percentage deviation between nominal and calculated concentrations, should fall within the 85% to 115% range (80% to 120% at LLOQ).

- 1. *Matrix effects*

For the determination of matrix effect, the peak area ratio of post-extracted blank matrix spiked with the analytes were compared with those in standard solutions at equivalent concentrations (both low and high). Each level of concentration samples was assessed by analyzing six samples at different sources. The precision should not be greater than 15%.

- 1. *Sample stability*

Stability assessments incorporated both surrogate and authentic analytes. The stability of authentic analyte in plasma was evaluated at low and high QC levels under various storage conditions, including room temperature for 2 h, three freeze-thaw cycles, 24 h incubation in an autosampler (6 °C), and long-term storage at −70 °C. Meanwhile, the concentration of each endogenous compound in the blank sample was determined and subtracted from the total concentration of the same compound observed in the corresponding spiked sample. The mean concentration at each QC level should vary by no more than ±15% from the nominal concentration. The stability of stock and working solutions was evaluated under conditions relevant to routine analysis. Stock solutions and working solutions prepared in methanol were stored at −70 °C and assessed after long-term storage. Stability was determined by comparing the measured concentrations of stored solutions with freshly prepared solutions at same levels. Solutions were considered stable if the concentration deviation was within ±15% of nominal.

- 1. *Recovery*

When a surrogate analyte approach is used, the recovery should be evaluated for both the native analytes and their surrogate analytes. Recovery should be assessed at low, medium, and high QC levels, using a comparison of pre-extraction spiked and post-extraction spiked samples. Recovery of the analyte does not need to be 100%, but the extent of recovery of an analyte and of the IS should be consistent. The recovery of the surrogate analyte should be comparable to that of the native analyte.

Table S1 Optimized multiple reaction monitoring parameters of the analytes and ISs in this study.

| Compound | Precursor ion species | Q1 Mass (Da) | Q3 Mass (Da) | DP (V) | CE (eV) |
| --- | --- | --- | --- | --- | --- |
| TCA | [M–H]^–^ | 514.3 | 80.0 | –100 | –130 |
| TCA-d4 | [M–H]^–^ | 518.2 | 80.0 | –60 | –130 |
| TUDCA | [M–H]^–^ | 498.2 | 80.1 | –120 | –130 |
| TUDCA-d4 | [M–H]^–^ | 502.3 | 80.1 | –120 | –130 |
| TCDCA | [M–H]^–^ | 498.3 | 80.1 | –120 | –130 |
| TCDCA-d4 | [M–H]^–^ | 502.2 | 80.1 | –120 | –130 |
| CA | [M–H]^–^ | 453.3 | 407.3 | –70 | –25 |
| CA-d4 | [M–H]^–^ | 457.3 | 411.3 | –60 | –20 |
| UDCA | [M–H]^–^ | 437.2 | 391.2 | –60 | –45 |
| UDCA-d4 | [M–H]^–^ | 441.2 | 395.2 | –60 | –45 |
| CDCA | [M–H]^–^ | 437.3 | 391.3 | –60 | –45 |
| CDCA-d4 | [M–H]^–^ | 441.3 | 395.3 | –60 | –45 |
| IS1 | [M–H]^–^ | 294.0 | 250.1 | –40 | –17 |
| IS2 | [M–H]^–^ | 269.1 | 170.0 | –80 | –24 |

Table S2 Response factors (RFs) in rat plasma.

| RF | Mean ± SEM (*n* = 12) | RSD (%) |
| --- | --- | --- |
| TCA-d4/TCA | 0.63±0.02 | 9.14 |
| TUDCA-d4/TUDCA | 0.62±0.01 | 3.87 |
| TCDCA-d4/TCDCA | 0.85±0.02 | 9.33 |
| CA-d4/CA | 1.59±0.02 | 5.22 |
| UDCA-d4/UDCA | 0.95±0.00 | 1.18 |
| CDCA-d4/CDCA | 0.66±0.00 | 1.77 |

Table S3 The calibration curves, determination coefficients (r) and linearity ranges of the investigated analytes.

| Compound | Regression equation | Linear range (ng/mL) | r | Weighting factor |
| --- | --- | --- | --- | --- |
| Analysis of bile acids | | | | |
| TCA-d4 | y=0.00252x-0.00517 | 20~14580 | 0.9992 | 1/x^2^ |
| TUDCA-d4 | y=0.00275x-0.00184 | 10~7290 | 0.9981 | 1/x^2^ |
| TCDCA-d4 | y=0.00311x-0.00161 | 10~7290 | 0.9987 | 1/x^2^ |
| CA-d4 | y=0.014x+0.00176 | 20~14580 | 0.9992 | 1/x^2^ |
| UDCA-d4 | y=0.0112x-0.00572 | 10~7290 | 0.9986 | 1/x^2^ |
| CDCA-d4 | y=0.00833x-0.00111 | 10~7290 | 0.9979 | 1/x^2^ |
| Analysis of deuterated bile acids | | | | |
| TCA-d4 | y=0.0637x+0.0812 | 0.3~243 | 0.9985 | 1/x^2^ |
| TUDCA-d4 | y=0.0833x-0.00796 | 0.3~243 | 0.9980 | 1/x^2^ |
| TCDCA-d4 | y=0.0882x-0.00565 | 0.3~243 | 0.9989 | 1/x^2^ |
| CA-d4 | y=0.768x+0.0104 | 0.3~243 | 0.9975 | 1/x^2^ |
| UDCA-d4 | y=0.532x+0.232 | 0.3~243 | 0.9985 | 1/x^2^ |
| CDCA-d4 | y=0.393x+0.118 | 0.3~243 | 0.9990 | 1/x^2^ |

Table S4 Accuracy and precision of six surrogate analytes in rat plasma for determination of bile acids (*n* = 6).

| Compound | Nominal concentration (ng/mL) | Intra-day (n = 6) | | | Inter-day (n = 18) | | |
| --- | --- | --- | --- | --- | --- | --- | --- |
|  |  | Measured concentration | Accuracy (RE, %) | Precision (RSD, %) | Measured concentration | Accuracy (RE, %) | Precision (RSD, %) |
|  |  | （ng/mL, Mean ± SEM） |  |  | （ng/mL, Mean ± SEM） |  |  |
| TCA-d4 | 20 | 19.2 ± 0.50 | 96.2 | 6.41 | 20.3 ± 0.39 | 102 | 8.12 |
|  | 60 | 61.6 ± 0.96 | 103 | 3.81 | 61.5 ± 0.43 | 102 | 2.95 |
|  | 540 | 557 ± 4.66 | 103 | 2.05 | 557 ± 2.69 | 103 | 2.04 |
|  | 11664 | 11517 ± 111 | 98.7 | 2.36 | 11406 ± 76.9 | 97.8 | 2.86 |
| TUDCA-d4 | 10 | 8.91 ± 0.27 | 89.1 | 7.35 | 10.1 ± 0.28 | 101 | 11.5 |
|  | 30 | 30.1 ± 0.66 | 100 | 5.38 | 30.7 ± 0.33 | 102 | 4.59 |
|  | 270 | 282 ± 3.46 | 105 | 3.00 | 282 ± 1.63 | 105 | 2.44 |
|  | 5832 | 5803 ± 51.0 | 99.5 | 2.15 | 5688 ± 36.0 | 97.5 | 2.69 |
| TCDCA-d4 | 10 | 9.68 ± 0.14 | 96.8 | 3.55 | 9.84 ± 0.22 | 98.4 | 9.67 |
|  | 30 | 30.5 ± 0.86 | 102 | 6.88 | 30.6 ± 0.40 | 102 | 5.49 |
|  | 270 | 283 ± 2.99 | 105 | 2.58 | 281 ± 1.44 | 104 | 2.17 |
|  | 5832 | 5690 ± 51.0 | 97.6 | 2.20 | 5641 ± 37.5 | 96.7 | 2.82 |
| CA-d4 | 20 | 19.2 ± 0.37 | 96.0 | 4.73 | 19.8 ± 0.33 | 98.9 | 7.13 |
|  | 60 | 58.4 ± 0.78 | 97.3 | 3.29 | 59.7 ± 0.45 | 100 | 3.21 |
|  | 540 | 548 ± 3.79 | 101 | 1.70 | 545 ± 2.56 | 101 | 1.99 |
|  | 11664 | 11767 ± 88.2 | 101 | 1.84 | 11667 ± 48.5 | 100 | 1.76 |
| UDCA-d4 | 10 | 9.36 ± 0.17 | 93.6 | 4.51 | 9.13 ± 0.16 | 91.3 | 7.56 |
|  | 30 | 30.7 ± 0.32 | 102 | 2.56 | 31.2 ± 0.31 | 104 | 4.15 |
|  | 270 | 279 ± 1.69 | 103 | 1.49 | 280 ± 1.09 | 104 | 1.65 |
|  | 5832 | 5588 ± 39.6 | 95.8 | 1.74 | 5576 ± 27.1 | 95.6 | 2.06 |
| CDCA-d4 | 10 | 10.1 ± 0.29 | 101 | 7.02 | 9.83 ± 0.19 | 98.3 | 8.13 |
|  | 30 | 31.9 ± 0.18 | 106 | 1.38 | 31.1 ± 0.27 | 104 | 3.73 |
|  | 270 | 283 ± 1.64 | 105 | 1.42 | 280 ± 1.45 | 104 | 2.19 |
|  | 5832 | 5432 ± 36.8 | 93.1 | 1.66 | 5421 ± 30.2 | 92.9 | 2.37 |

Table S5 Accuracy and precision of six analytes in rat plasma for determination of deuterated bile acids (*n* = 6).

| Compound | Nominal concentration (ng/mL) | Intra-day (n = 6) | | | Inter-day (n = 18) | | |
| --- | --- | --- | --- | --- | --- | --- | --- |
|  |  | Measured concentration | Accuracy (RE, %) | Precision (RSD, %) | Measured concentration | Accuracy (RE, %) | Precision (RSD, %) |
|  |  | （ng/mL, Mean ± SEM） |  |  | （ng/mL, Mean ± SEM） |  |  |
| TCA-d4 | 0.3 | 0.32 ± 0.01 | 107 | 4.73 | 0.33 ± 0.01 | 110 | 7.13 |
|  | 1 | 0.97 ± 0.01 | 97.3 | 3.29 | 1.00 ± 0.01 | 100 | 3.21 |
|  | 9 | 9.13 ± 0.06 | 101 | 1.70 | 9.09 ± 0.04 | 101 | 1.99 |
|  | 194.4 | 196 ± 1.47 | 101 | 1.84 | 194 ± 0.81 | 100 | 1.76 |
| TUDCA-d4 | 0.3 | 0.34 ± 0.01 | 113 | 8.64 | 0.32 ± 0.01 | 106 | 7.74 |
|  | 1 | 1.04 ± 0.01 | 104 | 3.15 | 1.03 ± 0.01 | 103 | 5.08 |
|  | 9 | 9.17 ± 0.08 | 102 | 2.12 | 9.15 ± 0.05 | 102 | 2.49 |
|  | 194.4 | 197 ± 1.39 | 101 | 1.73 | 196 ± 0.85 | 101 | 1.85 |
| TCDCA-d4 | 0.3 | 0.34 ± 0.01 | 112 | 6.79 | 0.34 ± 0.005 | 112 | 5.97 |
|  | 1 | 1.01 ± 0.01 | 101 | 2.30 | 1.01 ± 0.01 | 101 | 4.23 |
|  | 9 | 9.10 ± 0.11 | 101 | 3.01 | 9.08 ± 0.06 | 101 | 2.79 |
|  | 194.4 | 203 ± 1.93 | 104 | 2.34 | 200 ± 0.98 | 103 | 2.07 |
| CA-d4 | 0.3 | 0.34 ± 0.01 | 112 | 8.15 | 0.33 ± 0.01 | 111 | 7.76 |
|  | 1 | 1.01 ± 0.01 | 101 | 2.40 | 1.01 ± 0.01 | 101 | 2.66 |
|  | 9 | 9.11 ± 0.09 | 101 | 2.31 | 9.07 ± 0.06 | 101 | 2.16 |
|  | 194.4 | 194 ± 1.17 | 100 | 1.48 | 194 ± 0.91 | 100 | 1.63 |
| UDCA-d4 | 0.3 | 0.34 ± 0.01 | 113 | 5.47 | 0.34 ± 0.01 | 115 | 6.82 |
|  | 1 | 1.04 ± 0.01 | 104 | 2.19 | 1.03 ± 0.01 | 103 | 3.85 |
|  | 9 | 9.12 ± 0.06 | 101 | 1.69 | 9.15 ± 0.04 | 102 | 1.65 |
|  | 194.4 | 188 ± 1.65 | 96.6 | 2.15 | 187 ± 0.74 | 96.3 | 1.67 |
| CDCA-d4 | 0.3 | 0.34 ± 0.01 | 112 | 5.09 | 0.33 ± 0.005 | 110 | 5.93 |
|  | 1 | 1.04 ± 0.02 | 104 | 4.45 | 1.02 ± 0.01 | 102 | 3.85 |
|  | 9 | 9.21 ± 0.06 | 102 | 1.56 | 9.24 ± 0.05 | 103 | 2.16 |
|  | 194.4 | 187 ± 1.71 | 96.3 | 2.24 | 186 ± 0.83 | 95.7 | 1.90 |

Table S6 Matrix effect of six analytes in rat plasma (*n* = 6).

| Compound | Determination of BAs | | | Compound | Determination of DBAs | | |
| --- | --- | --- | --- | --- | --- | --- | --- |
|  | Nominal concentration  (ng/mL) | Matrix factor  (Mean ± SEM) | Matrix effect  （RSD, %） |  | Nominal concentration  (ng/mL) | Matrix factor  (Mean ± SEM) | Matrix effect  （RSD, %） |
| TCA | 60 | 0.97±0.02 | 4.57 | TCA-d4 | 1 | 1.00±0.01 | 3.10 |
|  | 11664 | 0.99±0.02 | 3.87 |  | 194.4 | 1.00±0.02 | 4.48 |
| TUDCA | 30 | 0.97±0.02 | 4.13 | TUDCA-d4 | 1 | 0.96±0.01 | 3.69 |
|  | 5832 | 1.00±0.02 | 4.07 |  | 194.4 | 1.02±0.02 | 4.92 |
| TCDCA | 30 | 1.05±0.00 | 0.97 | TCDCA-d4 | 1 | 1.01±0.01 | 1.51 |
|  | 5832 | 1.03±0.01 | 3.13 |  | 194.4 | 1.04±0.01 | 3.33 |
| CA | 60 | 0.98±0.02 | 3.87 | CA-d4 | 1 | 0.98±0.02 | 5.22 |
|  | 11664 | 0.99±0.02 | 5.98 |  | 194.4 | 1.00±0.02 | 4.36 |
| UDCA | 30 | 1.01±0.00 | 1.20 | UDCA-d4 | 1 | 0.97±0.01 | 2.15 |
|  | 5832 | 1.01±0.01 | 2.58 |  | 194.4 | 1.01±0.01 | 3.00 |
| CDCA | 30 | 1.03±0.02 | 4.40 | CDCA-d4 | 1 | 0.97±0.01 | 2.82 |
|  | 5832 | 1.05±0.01 | 3.36 |  | 194.4 | 1.04±0.01 | 2.15 |

Table S7 Stability of six analytes in rat plasma for determination of bile acids (*n* = 6).

| Compound | Nominal concentration (ng/mL) | Nominal concentration containing background (ng/mL) | Measured concentration (ng/mL, Mean ± SEM) | Bias, % | RSD, % |  |
| --- | --- | --- | --- | --- | --- | --- |
|  |  |  |  |  |  |  |
| 2 h, room temperature | | | | | |  |
| TCA | Background | — | 81.5 ± 1.21 | — | 3.64 |  |
|  | 60 | 142 | 134 ± 0.79 | –5.20 | 1.44 |  |
|  | 11664 | 11746 | 11248 ± 108 | –4.23 | 2.34 |  |
| TUDCA | Background | — | 24.4 ± 0.61 | — | 6.08 |  |
|  | 30 | 54.4 | 52.1 ± 1.05 | –4.31 | 4.93 |  |
|  | 5832 | 5856 | 5661 ± 63.5 | –3.33 | 2.75 |  |
| TCDCA | Background | — | 14.9 ± 0.60 | — | 9.96 |  |
|  | 30 | 44.9 | 41.5 ± 0.95 | –7.42 | 5.61 |  |
|  | 5832 | 5847 | 5802 ± 53.7 | –0.77 | 2.27 |  |
| CA | Background | — | 284 ± 2.15 | — | 1.85 |  |
|  | 60 | 344 | 354 ± 2.76 | 2.92 | 1.91 |  |
|  | 11664 | 11948 | 12232 ± 96.3 | 2.38 | 1.93 |  |
| UDCA | Background | — | 24.6 ± 0.22 | — | 2.22 |  |
|  | 30 | 54.6 | 53.9 ± 0.30 | –1.23 | 1.36 |  |
|  | 5832 | 5857 | 5999 ± 76.8 | 2.42 | 3.14 |  |
| CDCA | Background | — | 244.6 ± 2.84 | — | 2.85 |  |
|  | 30 | 275 | 276.8 ± 1.92 | 0.81 | 1.70 |  |
|  | 5832 | 6077 | 5919 ± 112 | –2.59 | 4.65 |  |
| 3 freeze–thaw cycles | | | | | |  |
| TCA | Background | — | 82.1 ± 0.91 | — | 2.73 |  |
|  | 60 | 142 | 138 ± 1.68 | –2.94 | 2.98 |  |
|  | 11664 | 11746 | 11408 ± 131 | –2.87 | 2.82 |  |
| TUDCA | Background | — | 25.0 ± 0.41 | — | 4.00 |  |
|  | 30 | 55.0 | 52.1 ± 1.08 | –5.31 | 5.08 |  |
|  | 5832 | 5857 | 5804 ± 89.3 | –0.90 | 3.77 |  |
| TCDCA | Background | — | 15.1 ± 0.90 | — | 14.5 |  |
|  | 30 | 45.1 | 42.0 ± 0.64 | –7.00 | 3.73 |  |
|  | 5832 | 5847 | 5826 ± 85.7 | –0.36 | 3.60 |  |
| CA | Background | — | 276 ± 1.63 | — | 1.44 |  |
|  | 60 | 336 | 302 ± 6.86 | –10.1 | 5.56 |  |
|  | 11664 | 11940 | 12401 ± 242 | 3.86 | 4.78 |  |
| UDCA | Background | — | 23.9 ± 0.27 | — | 2.77 |  |
|  | 30 | 53.9 | 54.4 ± 1.19 | 0.88 | 5.36 |  |
|  | 5832 | 5856 | 5906 ± 76.1 | 0.85 | 3.16 |  |
| CDCA | Background | — | 241 ± 3.37 | — | 3.42 |  |
|  | 30 | 271 | 280 ± 3.80 | 3.37 | 3.32 |  |
|  | 5832 | 6073 | 5923 ± 112 | –2.47 | 4.64 |  |
| 24 h, 6 °C | | | | | |  |
| TCA | Background | — | 1023 ± 34.8 | — | 3.41 |  |
|  | 60 | 1083 | 1092 ± 23.7 | 0.83 | 2.17 |  |
|  | 11664 | 12687 | 13647 ± 594 | 7.57 | 4.35 |  |
| TUDCA | Background | — | 104 ± 2.34 | — | 2.26 |  |
|  | 30 | 134 | 132 ± 2.56 | –1.10 | 1.94 |  |
|  | 5832 | 5936 | 6180 ± 193 | 4.11 | 3.12 |  |
| TCDCA | Background | — | 89.3 ± 2.76 | — | 3.09 |  |
|  | 30 | 119 | 120 ± 2.26 | 0.39 | 1.88 |  |
|  | 5832 | 5921 | 6244 ± 243 | 5.46 | 3.89 |  |
| CA | Background | — | 597 ± 16.5 | — | 2.77 |  |
|  | 60 | 657 | 663 ± 18.9 | 0.91 | 2.85 |  |
|  | 11664 | 12261 | 12462 ± 535 | 1.64 | 4.29 |  |
| UDCA | Background | — | 10.2 ± 0.21 | — | 2.05 |  |
|  | 30 | 40.2 | 40.7 ± 0.64 | 1.16 | 1.57 |  |
|  | 5832 | 5842 | 5684 ± 215 | –2.71 | 3.78 |  |
| CDCA | Background | — | 17.7 ± 0.73 | — | 4.09 |  |
|  | 30 | 47.7 | 47.4 ± 0.61 | –0.66 | 1.28 |  |
|  | 5832 | 5850 | 5665 ± 135 | –3.16 | 2.38 |  |
| 123 d, –70 ℃ | | | | | |  |
| TCA | Background | — | 142 ± 2.68 | — | 1.88 |  |
|  | 60 | 202 | 201 ± 4.84 | –0.56 | 2.41 |  |
|  | 11664 | 11806 | 12196 ± 400 | 3.30 | 3.28 |  |
| TUDCA | Background | — | 35.5 ± 1.85 | — | 5.22 |  |
|  | 30 | 65.5 | 65.2 ± 1.00 | –0.52 | 1.53 |  |
|  | 5832 | 5867 | 6001 ± 235 | 2.27 | 3.92 |  |
| TCDCA | Background | — | 113 ± 3.36 | — | 2.98 |  |
|  | 30 | 143 | 141 ± 7.88 | –1.66 | 5.61 |  |
|  | 5832 | 5945 | 6241 ± 237 | 4.98 | 3.80 |  |
| CA | Background | — | 149 ± 7.12 | — | 4.78 |  |
|  | 60 | 209 | 196 ± 9.87 | –6.12 | 5.04 |  |
|  | 11664 | 11813 | 11900 ± 308 | 0.74 | 2.59 |  |
| UDCA | Background | — | 35.3 ± 1.18 | — | 3.34 |  |
|  | 30 | 65.3 | 65.3 ± 2.61 | –0.05 | 4.00 |  |
|  | 5832 | 5867 | 5977 ± 216 | 1.86 | 3.62 |  |
| CDCA | Background | — | 74.6 ± 2.03 | — | 2.72 |  |
|  | 30 | 105 | 104 ± 5.96 | –0.35 | 5.72 |  |
|  | 5832 | 5907 | 5754 ± 246 | –2.59 | 4.27 |  |

—: no data.

Table S8 Stability of six analytes in rat plasma for determination of deuterated bile acids (*n* = 6).

| Compound | Nominal concentration (ng/mL) | Measured concentration (ng/mL, Mean ± SEM) | Bias, % | RSD, % |  |
| --- | --- | --- | --- | --- | --- |
|  |  |  |  |  |  |
| 2 h, room temperature | | | | |  |
| TCA-d4 | 1 | 1.00 ± 0.01 | 0.17 | 3.52 |  |
|  | 194.4 | 194 ± 1.91 | –0.26 | 2.41 |  |
| TUDCA-d4 | 1 | 1.04 ± 0.01 | 4.44 | 2.20 |  |
|  | 194.4 | 193 ± 2.17 | –0.61 | 2.75 |  |
| TCDCA-d4 | 1 | 1.01 ± 0.02 | 0.72 | 3.71 |  |
|  | 194.4 | 200 ± 1.59 | 2.88 | 1.94 |  |
| CA-d4 | 1 | 1.01 ± 0.01 | 0.56 | 1.27 |  |
|  | 194.4 | 199 ± 2.12 | 2.16 | 2.62 |  |
| UDCA-d4 | 1 | 1.02 ± 0.01 | 2.00 | 2.65 |  |
|  | 194.4 | 188 ± 1.98 | –3.52 | 2.59 |  |
| CDCA-d4 | 1 | 1.02 ± 0.01 | 2.33 | 2.44 |  |
|  | 194.4 | 184 ± 2.42 | –5.35 | 3.23 |  |
| 3 freeze–thaw cycles | | | | |  |
| TCA-d4 | 1 | 1.04 ± 0.01 | 3.70 | 3.05 |  |
|  | 194.4 | 203 ± 0.78 | 4.63 | 0.94 |  |
| TUDCA-d4 | 1 | 1.04 ± 0.01 | 4.07 | 2.16 |  |
|  | 194.4 | 195 ± 1.21 | 0.31 | 1.52 |  |
| TCDCA-d4 | 1 | 1.03 ± 0.01 | 2.84 | 1.59 |  |
|  | 194.4 | 198 ± 2.46 | 1.60 | 3.05 |  |
| CA-d4 | 1 | 0.99 ± 0.01 | –0.61 | 1.42 |  |
|  | 194.4 | 177 ± 1.38 | –8.94 | 1.91 |  |
| UDCA-d4 | 1 | 1.02 ± 0.01 | 2.38 | 1.44 |  |
|  | 194.4 | 205 ± 2.24 | 5.24 | 2.68 |  |
| CDCA-d4 | 1 | 1.06 ± 0.01 | 6.20 | 1.86 |  |
|  | 194.4 | 201 ± 1.54 | 3.60 | 1.88 |  |
| 24 h, 6 °C | | | | |  |
| TCA-d4 | 1 | 1.01 ± 0.02 | 1.25 | 4.83 |  |
|  | 194.4 | 198 ± 1.72 | 2.02 | 2.13 |  |
| TUDCA-d4 | 1 | 1.05 ± 0.02 | 5.17 | 4.30 |  |
|  | 194.4 | 195 ± 1.71 | 0.45 | 2.14 |  |
| TCDCA-d4 | 1 | 1.07 ± 0.02 | 6.83 | 4.00 |  |
|  | 194.4 | 195 ± 2.16 | 0.34 | 2.72 |  |
| CA-d4 | 1 | 1.05 ± 0.01 | 5.25 | 1.85 |  |
|  | 194.4 | 205 ± 2.20 | 5.25 | 2.63 |  |
| UDCA-d4 | 1 | 1.05 ± 0.01 | 4.94 | 2.94 |  |
|  | 194.4 | 189 ± 1.62 | –2.98 | 2.10 |  |
| CDCA-d4 | 1 | 1.04 ± 0.02 | 4.00 | 4.19 |  |
|  | 194.4 | 185 ± 2.05 | –4.89 | 2.72 |  |
| 123 d, –70 ℃ | | | | |  |
| TCA–d4 | 1 | 0.95 ± 0.01 | –4.66 | 3.18 |  |
|  | 194.4 | 185 ± 4.11 | –4.86 | 5.44 |  |
| TUDCA–d4 | 1 | 1.01 ± 0.02 | 0.76 | 4.29 |  |
|  | 194.4 | 179 ± 1.69 | –8.03 | 2.32 |  |
| TCDCA–d4 | 1 | 0.97 ± 0.02 | –2.58 | 4.14 |  |
|  | 194.4 | 204 ± 3.32 | 5.05 | 3.98 |  |
| CA–d4 | 1 | 0.97 ± 0.01 | –3.02 | 3.57 |  |
|  | 194.4 | 197 ± 3.78 | 1.27 | 4.70 |  |
| UDCA–d4 | 1 | 0.99 ± 0.01 | –0.98 | 1.99 |  |
|  | 194.4 | 187 ± 1.60 | –3.65 | 2.09 |  |
| CDCA–d4 | 1 | 1.03 ± 0.02 | 3.43 | 3.69 |  |
|  | 194.4 | 190 ± 1.31 | –2.27 | 1.68 |  |

Table S9 Stability of stock solutions stored at –70 ℃ in 112 days (*n* = 6).

| Compound | day | Nominal concentration (ng/mL) | Peak area (Mean ± SEM) | Bias, % | RSD, % |
| --- | --- | --- | --- | --- | --- |
| TCA-d4 | 0 | 1,000,000 | 1034667 ± 14847 | –0.32 | 3.51 |
|  | 112 |  | 1031333 ± 10414 |  | 2.47 |
| TUDCA-d4 | 0 | 1,000,000 | 1075000 ± 12845 | –1.71 | 2.93 |
|  | 112 |  | 1056667 ± 14298 |  | 3.31 |
| TCDCA-d4 | 0 | 1,000,000 | 1278333 ± 17208 | 9.13 | 3.30 |
|  | 112 |  | 1395000 ± 15864 |  | 2.79 |
| CA-d4 | 0 | 1,000,000 | 1106000 ± 13023 | 7.44 | 2.88 |
|  | 112 |  | 1188333 ± 25453 |  | 5.25 |
| UDCA-d4 | 0 | 1,000,000 | 6075000 ± 115261 | 8.70 | 4.65 |
|  | 112 |  | 6603333 ± 98308 |  | 3.65 |
| CDCA-d4 | 0 | 1,000,000 | 5760000 ± 72938 | 3.13 | 3.10 |
|  | 112 |  | 5940000 ± 99331 |  | 4.10 |
| IS1 | 0 | 1,000,000 | 4583333 ± 53020 | 0.51 | 2.83 |
|  | 112 |  | 4606667 ± 36025 |  | 1.92 |
| IS2 | 0 | 1,000,000 | 5618333 ± 137269 | –3.41 | 5.98 |
|  | 112 |  | 5426667 ± 123980 |  | 5.60 |

Table S10 Stability of work solutions stored at –70 ℃ in 21 days (*n* = 6).

| Compound | day | Determination of BAs | | | | Determination of deuterated BAs | | | |
| --- | --- | --- | --- | --- | --- | --- | --- | --- | --- |
|  |  | Nominal concentration (ng/mL) | Peak area (Mean ± SEM) | Bias, % | RSD, % | Nominal concentration (ng/mL) | Peak area (Mean ± SEM) | Bias, % | RSD, % |
| TCA-d4 | 0 | 200 | 39950 ± 830 | 1.13 | 5.09 | 3 | 5768 ± 84.9 | –0.92 | 3.61 |
|  | 21 |  | 40400 ± 493 |  | 2.99 |  | 5715 ± 110 |  | 4.71 |
|  | 0 | 145,800 | 11800000 ± 106458 | –1.84 | 2.21 | 2,430 | 193667 ± 3739 | 1.20 | 4.73 |
|  | 21 |  | 11583333 ± 257445 |  | 5.44 |  | 196000 ± 2781 |  | 3.48 |
| TUDCA-d4 | 0 | 100 | 23883 ± 691 | 0.14 | 7.09 | 3 | 7838 ± 201 | 3.27 | 6.28 |
|  | 21 |  | 23917 ± 192 |  | 1.97 |  | 8095 ± 103 |  | 3.11 |
|  | 0 | 72,900 | 7020000 ± 25820 | –1.33 | 0.90 | 2,430 | 236167 ± 1956 | –3.25 | 2.03 |
|  | 21 |  | 6926667 ± 131064 |  | 4.63 |  | 228500 ± 3462 |  | 3.71 |
| TCDCA-d4 | 0 | 100 | 31150 ± 595 | 2.19 | 4.68 | 3 | 10555 ± 161 | –1.04 | 3.73 |
|  | 21 |  | 31833 ± 387 |  | 2.98 |  | 10445 ± 182 |  | 4.27 |
|  | 0 | 72,900 | 9351667 ± 78588 | –1.80 | 2.06 | 2,430 | 305000 ± 4844 | 2.57 | 3.89 |
|  | 21 |  | 9183333 ± 165362 |  | 4.41 |  | 312833 ± 3400 |  | 2.66 |
| CA-d4 | 0 | 200 | 43300 ± 306 | –1.54 | 1.73 | 3 | 6067 ± 89.6 | 2.34 | 3.62 |
|  | 21 |  | 42633 ± 1070 |  | 6.15 |  | 6208 ± 128 |  | 5.03 |
|  | 0 | 145,800 | 12183333 ± 100852 | 2.52 | 2.03 | 2,430 | 204833 ± 1470 | 0.65 | 1.76 |
|  | 21 |  | 12490000 ± 92195 |  | 1.81 |  | 206167 ± 2386 |  | 2.84 |
| UDCA-d4 | 0 | 100 | 93367 ± 1804 | 1.48 | 4.73 | 3 | 30883 ± 539 | 3.08 | 4.28 |
|  | 21 |  | 94750 ± 620 |  | 1.60 |  | 31833 ± 196 |  | 1.51 |
|  | 0 | 72,900 | 25066667 ± 172562 | –1.40 | 1.69 | 2,430 | 821667 ± 10455 | 1.99 | 3.12 |
|  | 21 |  | 24716667 ± 345848 |  | 3.43 |  | 838000 ± 6763 |  | 1.98 |
| CDCA-d4 | 0 | 100 | 89817 ± 896 | 1.22 | 2.44 | 3 | 30250 ± 201 | –0.94 | 1.63 |
|  | 21 |  | 90917 ± 411 |  | 1.11 |  | 29967 ± 276 |  | 2.26 |
|  | 0 | 72,900 | 22566667 ± 122927 | –1.03 | 1.33 | 2,430 | 742667 ± 7154 | 1.53 | 2.36 |
|  | 21 |  | 22333333 ± 269155 |  | 2.95 |  | 754000 ± 6325 |  | 2.05 |
| IS2 | 0 | — | — | — | — | 100,000 | 5335000 ± 116125 | 7.03 | 5.33 |
|  | 21 |  | — |  | — |  | 5710000 ± 101686 |  | 4.36 |

Table S11 Recovery for true analyte, surrogate analyte, and IS1 for determination of bile acids (*n* = 6).

| Compound | Nominal concentration (ng/mL) | Recovery（%, Mean ± SEM） | RSD, % | Compound | Recovery（%, Mean ± SEM） | RSD, % |
| --- | --- | --- | --- | --- | --- | --- |
| TCA | 60 | 98.2±1.56 | 3.89 | TCA-d4 | 109 ± 1.80 | 4.04 |
|  | 540 | 97.8±1.37 | 3.43 |  | 93.4 ± 1.90 | 4.99 |
|  | 11664 | 101±0.95 | 2.30 |  | 109 ± 0.76 | 1.71 |
| TUDCA | 30 | 95.6±1.28 | 3.28 | TUDCA-d4 | 111 ± 1.67 | 3.67 |
|  | 270 | 105±1.48 | 3.45 |  | 94.1 ± 1.24 | 3.22 |
|  | 5832 | 103±1.11 | 2.64 |  | 114 ± 1.02 | 2.21 |
| TCDCA | 30 | 109±0.98 | 2.20 | TCDCA-d4 | 110 ± 1.25 | 2.79 |
|  | 270 | 108±1.29 | 2.93 |  | 92.0 ± 1.82 | 4.85 |
|  | 5832 | 105±0.93 | 2.17 |  | 113 ± 0.84 | 1.82 |
| CA | 60 | 110±0.86 | 1.92 | CA-d4 | 103 ± 1.19 | 2.84 |
|  | 540 | 99.4±1.74 | 4.29 |  | 101 ± 1.48 | 3.60 |
|  | 11664 | 94.8±0.69 | 1.78 |  | 100 ± 0.73 | 1.79 |
| UDCA | 30 | 111±2.10 | 4.63 | UDCA-d4 | 108 ± 1.11 | 2.52 |
|  | 270 | 102±1.53 | 3.67 |  | 103 ± 1.96 | 4.67 |
|  | 5832 | 96.3±0.52 | 1.32 |  | 106 ± 0.76 | 1.75 |
| CDCA | 30 | 103±1.47 | 3.50 | CDCA-d4 | 105 ± 1.23 | 2.89 |
|  | 270 | 106±1.59 | 3.67 |  | 104 ± 1.54 | 3.61 |
|  | 5832 | 104±0.82 | 1.93 |  | 102 ± 0.70 | 1.68 |
| IS1 | — | 97.8 ± 0.89 | 2.23 | — | — | — |

Table S12 PK parameters of six BAs and six DBAs in rats after oral administration of GBP+3DBAs.

| PK parameters^a^ | Total BAs | | | | | | DBAs | | | | | | Background^b^ | | | | | |
| --- | --- | --- | --- | --- | --- | --- | --- | --- | --- | --- | --- | --- | --- | --- | --- | --- | --- | --- |
|  | TCA | | TUDCA | | TCDCA | | TCA-d4 | | TUDCA-d4 | | TCDCA-d4 | | TCA | | TUDCA | | TCDCA | |
|  | ♂ | ♀ | ♂ | ♀ | ♂ | ♀ | ♂ | ♀ | ♂ | ♀ | ♂ | ♀ | ♂ | ♀ | ♂ | ♀ | ♂ | ♀ |
| Day1 | | | | | | | | | | | | | | | | | | |
| *C*_max_ (nmol/L) | 702±299 | 7988±1197^**^ | 554±149 | 379±66.1 | 339±66.2 | 2214±155^**^ | 15.7±5.54 | 53.5±8.86^*^ | 21.7±16.3 | 29.7±6.05 | 22.3±4.74 | 58.5±18.5 | 616±294 | 7972±1198^**^ | 427±140.3 | 243±53.6 | 217±24.3 | 2189±165^**^ |
| *T*_max_ (h) | 6.50±0.50 | 0.17 | 4.54±1.46 | 1.50±0.29 | 4.54±1.46 | 1.08 | 4.54±1.46 | 19.0±3.79^*^ | 3.54±1.47 | 1.04±0.37 | 7.00±0.58 | 6.50±1.50 | 9.04±5.17 | 0.17 | 6.50±0.50 | 0.17 | 5.04±1.69 | 1.09 |
| AUC_0-24 h_ (nmol∙h/L) | 6842±1278 | 108991±21131^*^ | 4958±561 | 4967±1104 | 3348±399 | 23391±2978^**^ | 114±23.4 | 900±159^*^ | 35.2±7.99 | 283±68.4^*^ | 173±58.9 | 834±286 | 5469±1441 | 100831±19789^*^ | 4421±562 | 2400±545^*^ | 2553±393 | 15687±545^**^ |
| AUC_0-∞_ (nmol∙h/L) | 9047 | 197698±59876 | 7150±871 | 7684±1692 | 4563±700 | 35981±6201^*^ | 131 | 1473 | 49.7±12.0 | 424±77.5^*^ | 243 | 1333±577 | 7406±1310 | 151095±47669 | 6608±726 | 4390±899 | 3776±922 | 21795±3806^*^ |
| *t*_1/2_ (h) | 12.6 | 8.78±4.28 | 12.1±3.11 | 12.7±3.73 | 8.43±0.84 | 12.3±5.00 | 6.51 | 4.27 | 3.87±1.38 | 11.7±1.55^**^ | 3.29 | 12.1±2.19^*^ | 7.51±4.89 | 6.74±3.36 | 10.0±3.99 | 19.7±6.61 | 8.08±2.11 | 8.40±3.70 |
| MRT (h) | 12.3±0.31 | 12.8±0.32 | 11.4±0.41 | 11.1±0.35 | 10.3±0.36 | 10.6±0.20 | 9.89±2.25 | 13.8±0.25 | 3.88±0.70 | 10.6±0.33^**^ | 8.22±0.99 | 11.8±0.57^*^ | 12.1±0.55 | 12.8±0.33 | 11.8±0.27 | 11.6±0.31 | 10.8±0.48 | 10.2±0.58 |
| Day7 | | | | | | | | | | | | | | | | | | |
| *C*_max_ (nmol/L) | 2106±433 | 9442±1971^*^ | 1628±533 | 1642±366^#^ | 1129±264 | 3170±430^**^ | 31.4±3.93 | 107±16.3^*^ | 24.2±5.76 | 50.5±9.10 | 59.9±9.63^#^ | 102±23.8 | 1822±403 | 8528±1793^*^ | 1442±509 | 1196±277 | 769±137^#^ | 2659±219^**^ |
| *T*_max_ (h) | 16.5±4.92 | 5.50±0.96 | 8.50±5.19 | 5.00±1.29 | 7.50±4.19 | 5.00±1.29^#^ | 12.5±5.56 | 7.50±0.50 | 2.54±0.92 | 6.00±1.41^#^ | 3.25±1.11^#^ | 7.50±0.50^*^ | 16.5±4.92 | 5.50±0.96 | 12.5±5.56 | 5.00±1.29 | 7.50±4.19 | 5.00±1.29^#^ |
| AUC_0-24 h_ (nmol∙h/L) | 27908±8465 | 120280±13280^**^ | 21311±4147**^#^** | 17024±2562^#^ | 13983±2285^#^ | 36078±732^**#^ | 502±70.4^#^ | 1552±108^**#^ | 200±25.7^##^ | 519±59.1^**#^ | 565±170 | 802±101 | 23380±7830 | 106161±12430^**^ | 19438±4230^#^ | 12254±2207^#^ | 8601±1102^#^ | 26618±1368^**##^ |
| AUC_0-∞_ (nmol∙h/L) | 32030±10697 | 199439±43571^*^ | 28681±6959^#^ | 23218±4578 | 19828±2465^#^ | 49810±5918^**^ | 1009±395^#^ | 2597±434^*^ | 354±78.8^#^ | 756±180 | 680±272 | 940±109 | 36115±9809 | 185863±28684^**^ | 26442±6981 | 17854±3651 | 9429±1106 | 42035±12440 |
| *t*_1/2_ (h) | 2.31±0.65 | 14.4±4.52 | 8.37±2.44 | 11.4±3.21 | 9.99±5.35 | 11.1±3.04 | 20.7±18.4 | 15.0±6.43 | 16.4±3.89 | 11.6±3.72 | 6.40±2.87 | 5.54±0.55^#^ | 11.1±9.43 | 18.1±5.13 | 8.65±2.73 | 10.5±4.63 | 1.47±0.25 | 12.3±7.79 |
| MRT (h) | 14.2±0.86 | 12.2±0.30 | 12.7±0.75 | 11.4±0.68 | 12.0±0.53^#^ | 10.6±0.64 | 13.7±0.52 | 12.9±0.23^#^ | 10.6±0.55^##^ | 11.1±0.34 | 7.89±1.21 | 9.97±1.62 | 14.2±0.94 | 12.1±0.30 | 12.9±0.79 | 11.5±0.80 | 13.7±1.29 | 10.7±0.60 |
| PK parameters^a^ | CA | | UDCA | | CDCA | | CA-d4 | | UDCA-d4 | | CDCA-d4 | | CA | | UDCA | | CDCA | |
|  | ♂ | ♀ | ♂ | ♀ | ♂ | ♀ | ♂ | ♀ | ♂ | ♀ | ♂ | ♀ | ♂ | ♀ | ♂ | ♀ | ♂ | ♀ |
| Day1 | | | | | | | | | | | | | | | | | | |
| *C*_max_ (nmol/L) | 18121±10448 | 3043±2626 | 1743±1020 | 112±57.6 | 3752±2553 | 325±178 | 59.0±23.9 | 7.50±3.86 | 123±72.9 | 10.8 | 214±114 | 129±44.2 | 17455±10182 | 3001±2583 | 649±358 | 70.1±16.5 | 2255±1566 | 174.5±74.6 |
| *T*_max_ (h) | 13.0±5.30 | 17.0±4.43 | 9.00±5.10 | 3.09±1.87 | 9.00±5.10 | 1.45±1.11 | 13.0±5.32 | 1.45±1.28 | 9.00±5.07 | 6.00 | 4.50±1.50 | 5.00±1.00 | 13.0±5.32 | 17.0±4.43 | 3.04±1.26 | 1.13±0.96 | 13.5±6.15 | 12.1 |
| AUC_0-24 h_ (nmol∙h/L) | 131389±58971 | 13027±8677 | 8444±2971 | 481±133 | 20006±9201 | 1794±543 | 508±114 | 10.6±10.3^*^ | 543±195 | 30.2 | 1564±478 | 732±213 | 126328±57226 | 12808±8654 | 3700±1245 | 382±64.8 | 15242±6784 | 1018±563 |
| AUC_0-∞_ (nmol∙h/L) | 175863±75198 | 39901 | 13025±1888 | 925 | 30403 | 2789 | 655 | — | 803±104 | — | 1654±513 | 764±216 | 171065±74531 | 15892±8345 | 5356±2027 | 467±56.6 | 21549 | 1068 |
| *t*_1/2_ (h) | 4.79±0.37 | 0.59 | 6.86±3.32 | 1.20 | 4.00 | 3.76 | 4.68 | — | 8.52±4.63 | — | 4.57±0.38 | 6.69±1.99 | 4.91±0.41 | 3.86±2.67 | 7.94±4.61 | 7.65±0.97 | 4.77 | 6.14 |
| MRT (h) | 13.9±2.82 | 13.2±2.39 | 9.20±2.10 | 3.90±0.07 | 10.5±2.20 | 7.90± 1.86 | 13.4±2.10 | 0.93±0.76^**^ | 11.6±4.34 | 5.85 | 8.7±1.88 | 7.93±1.19 | 13.9±2.80 | 14.0±2.63 | 9.63±2.12 | 10.3±1.07 | 12.5±1.99 | 7.29±2.90 |
| Day7 | | | | | | | | | | | | | | | | | | |
| *C*_max_ (nmol/L) | 13933±4082 | 31498±7910 | 2024±513 | 2739±901 | 2882±697 | 4967± 1628 | 60.9±12.8 | 63.9±10.7^#^ | 130±30.7 | 64.3±24.2 | 185±63.2 | 197±42.2 | 13443±4048 | 30914±7840 | 951±258 | 2262±767 | 2203±588 | 4511±1429 |
| *T*_max_ (h) | 3.00 | 6.75±5.75 | 2.00±0.41 | 6.75±5.75 | 2.50±0.29 | 0.79±0.21^**^ | 2.75±0.48 | 12.8±6.50 | 2.00±0.41 | 7.75±5.45 | 2.63±1.18 | 5.25±0.75 | 3.00 | 6.75±5.75 | 2.06±1.11 | 0.79±0.21 | 1.72±0.83 | 0.79±0.21 |
| AUC_0-24 h_ (nmol∙h/L) | 75200±18232 | 91417±15470^#^ | 10390±2253 | 8557±1682^#^ | 15614±3912 | 15036±4112 | 641±126 | 309±21.6^##^ | 528±125 | 269±66.5 | 1253±481 | 1126±278 | 69867±17251 | 88441±15123^#^ | 5634±1218 | 6515±1368^#^ | 14112±4082 | 14996±3936^#^ |
| AUC_0-∞_ (nmol∙h/L) | 67072±17704 | 136548±15089^*^ | 12725±3384 | 12388±1094 | 21063±9073 | 19647±2789^#^ | 871±396 | 768±109 | 653±137 | 344±95.2 | 1318±496 | 1235±372 | 74962±17255 | 112621±20228^#^ | 7045±2004 | 7241±1348^#^ | 18577±3062 | 19951±5447 |
| *t*_1/2_ (h) | 1.67±1.07 | 3.60±2.46 | 3.42±2.18 | 6.76±0.99 | 5.71±4.97 | 6.43±3.98 | 6.77±5.29 | 9.81±2.39 | 5.47±2.63 | 4.01±2.63 | 6.38±1.90 | 3.69±0.48 | 1.48±0.84 | 2.78±1.90 | 3.60±2.09 | 3.73±1.60 | 9.89±6.05 | 8.99±2.24 |
| MRT (h) | 11.4±1.67 | 10.0±2.70 | 8.54±0.66 | 7.36±2.46 | 9.33±0.86 | 6.98±1.70 | 12.1±0.56 | 12.6±2.03^#^ | 7.94±0.64 | 11.5±1.82 | 7.46±0.71 | 7.60±0.99 | 11.3±1.64 | 9.89±2.71 | 9.09±0.73 | 6.39±2.24 | 9.48±0.91 | 7.31±1.25 |

^a^ *C*_max_, peak concentration; *T*_max_, time to peak concentration; AUC_0-24 h_, area under the curve from 0 to 24 h; AUC_0-∞_, area under the curve from 0 extrapolated to infinite time; *t*_1/2_, elimination half life; MRT, mean residence time. ^*^*P* < 0.05, ^**^*P* < 0.01 *vs.* Male/Female; ^#^*P* < 0.05, ^##^*P* < 0.01 *vs.* Day 1/Day 7; —, data cannot be calculated, because half-lives were not estimable.

Table S13 Sensitivity analysis of endogenous BA AUC (nmol∙h/L) estimates under different scaling assumptions. Ranges represent endogenous AUC values calculated assuming scaling factors from 8× to 10×.

| Compound | Day1 | | Day7 | |
| --- | --- | --- | --- | --- |
|  | ♂ | ♀ | ♂ | ♀ |
| TCA | 5372~5565 | 99925~101738 | 22877~23883 | 104592~107731 |
| TUDCA | 4371~4481 | 2124~2684 | 19229~19646 | 11721~12786 |
| TCDCA | 2316~2607 | 14896~16554 | 8300~9300 | 25507~27716 |
| CA | 125766~126891 | 12816~12835 | 69940~70992 | 88111~88772 |
| UDCA | 3206~4268 | 352~419 | 5155~6227 | 6351~6812 |
| CDCA | 14791~15973 | 953~476 | 13312~15186 | 14170~13094 |


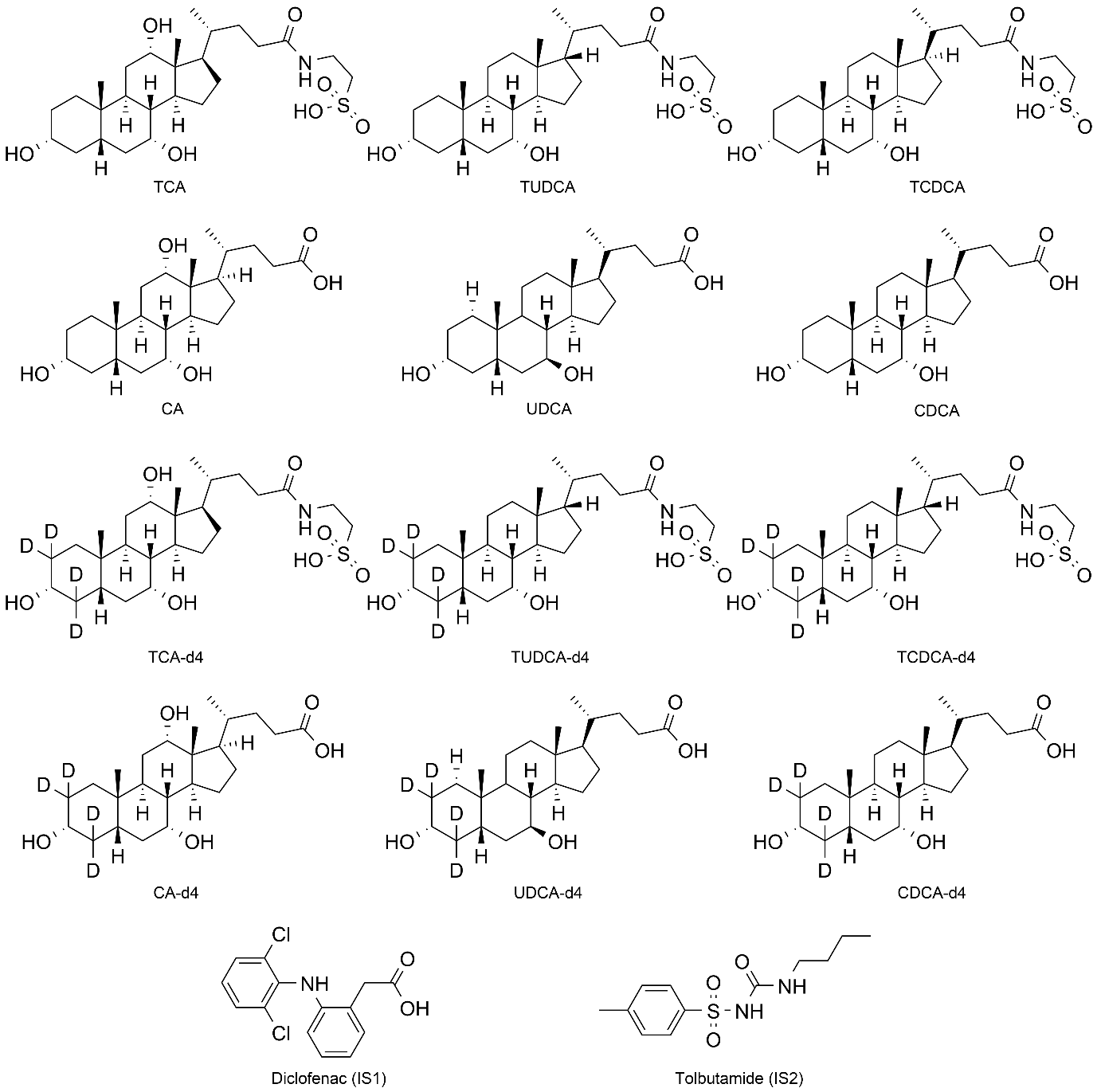


Figure S1 The structures of six BAs, six DBAs and two ISs.


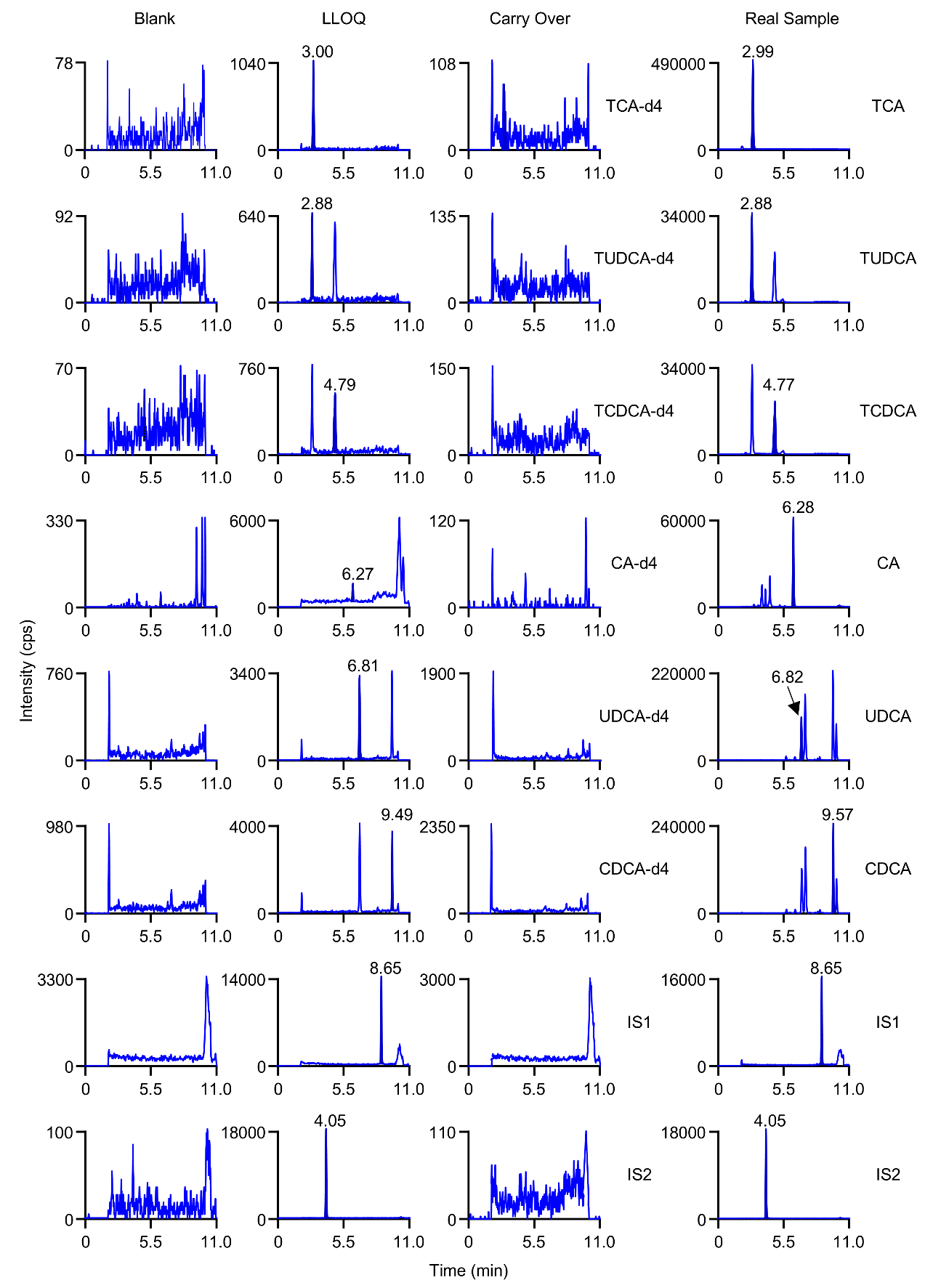


Figure S2 Chromatograms of six BAs, six DBAs and two ISs in rat plasma. Blank, blank plasma; LLOQ, lower limit of quantification; carry over, blank sample following the upper limit of quantification sample; real sample, the plasma sample after oral administration of GBP+3DBAs at the 1 h time point.


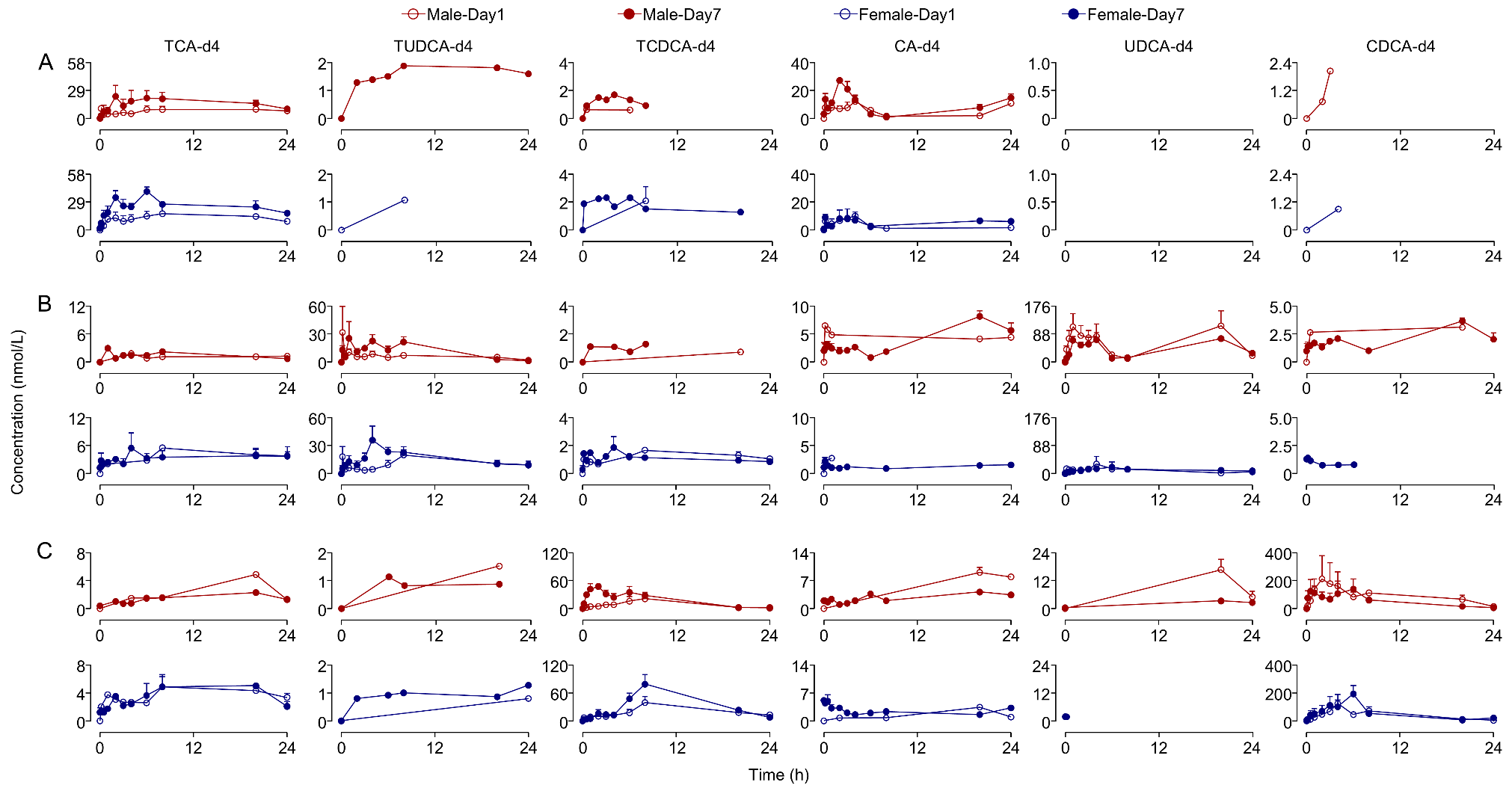


Figure S3 The mean plasma concentration–time profiles of six DBAs in rat plasma after oral administration of three taurine-conjugated DBAs. Data represent mean ± SEM (*n* = 3). (A) TCA-d4 group; (B) TUDCA-d4 group; (C) TCDCA-d4 group.
